# The potential of swine pseudorabies virus attenuated vaccine for oncolytic therapy against malignant tumors

**DOI:** 10.1186/s13046-023-02848-1

**Published:** 2023-10-27

**Authors:** Guosong Wang, Jiali Cao, Mengxuan Gui, Pengfei Huang, Liang Zhang, Ruoyao Qi, Ruiqi Chen, Lina Lin, Qiangyuan Han, Yanhua Lin, Tian Chen, Peiqing He, Jian Ma, Rao Fu, Junping Hong, Qian Wu, Hai Yu, Junyu Chen, Chenghao Huang, Tianying Zhang, Quan Yuan, Jun Zhang, Yixin Chen, Ningshao Xia

**Affiliations:** 1 State Key Laboratory of Vaccines for Infectious Diseases, National Institute of Diagnostics and Vaccine Development in Infectious Diseases, State Key Laboratory of Molecular Vaccinology and Molecular Diagnostics, Collaborative Innovation Center of Biologic ProductsNational Innovation Platform for Industry-Education Intergration in Vaccine ResearchSchool of Life Sciences, School of Public Health, Xiang An Biomedicine Laboratory, Xiamen University, Xiamen, People’s Republic of China; 2 Department of Laboratory Medicine, Fujian Key Clinical Specialty of Laboratory Medicine, Women and Children’s Hospital, School of Medicine, Xiamen University, Xiamen, People’s Republic of China

**Keywords:** Cancer therapy, Oncolytic virus, Pseudorabies virus, EGFR, Immune checkpoint

## Abstract

**Background:**

Oncolytic viruses are now well recognized as potential immunotherapeutic agents against cancer. However, the first FDA-approved oncolytic herpes simplex virus 1 (HSV-1), T-VEC, showed limited benefits in some patients in clinical trials. Thus, the identification of novel oncolytic viruses that can strengthen oncolytic virus therapy is warranted. Here, we identified a live-attenuated swine pseudorabies virus (PRV-LAV) as a promising oncolytic agent with broad-spectrum antitumor activity in vitro and in vivo.

**Methods:**

PRV cytotoxicity against tumor cells and normal cells was tested in vitro using a CCK8 cell viability assay. A cell kinase inhibitor library was used to screen for key targets that affect the proliferation of PRV-LAV. The potential therapeutic efficacy of PRV-LAV was tested against syngeneic tumors in immunocompetent mice, and against subcutaneous xenografts of human cancer cell lines in nude mice. Cytometry by time of flight (CyTOF) and flow cytometry were used to uncover the immunological mechanism of PRV-LAV treatment in regulating the tumor immune microenvironment.

**Results:**

Through various tumor-specific analyses, we show that PRV-LAV infects cancer cells via the NRP1/EGFR signaling pathway, which is commonly overexpressed in cancer. Further, we show that PRV-LAV kills cancer cells by inducing endoplasmic reticulum (ER) stress. Moreover, PRV-LAV is responsible for reprogramming the tumor microenvironment from immunologically naïve (“cold”) to inflamed (“hot”), thereby increasing immune cell infiltration and restoring CD8^+^ T cell function against cancer. When delivered in combination with immune checkpoint inhibitors (ICIs), the anti-tumor response is augmented, suggestive of synergistic activity.

**Conclusions:**

PRV-LAV can infect cancer cells via NRP1/EGFR signaling and induce cancer cells apoptosis via ER stress. PRV-LAV treatment also restores CD8^+^ T cell function against cancer. The combination of PRV-LAV and immune checkpoint inhibitors has a significant synergistic effect. Overall, these findings point to PRV-LAV as a serious potential candidate for the treatment of NRP1/EGFR pathway-associated tumors.

**Supplementary Information:**

The online version contains supplementary material available at 10.1186/s13046-023-02848-1.

## Introduction

Cancer is an increasingly serious public health problem globally [1]. An estimated 19.3 million new cancer cases and 10 million cancer deaths were projected to occur in 2020 [2]. According to statistics from the American Cancer Society, the number of cancer cases diagnosed annually is expected to increase to 23.6 million by 2030 [3]. In light of these statistics, new cancer drugs are urgently needed. Recent studies have identified oncolytic viruses as a relatively new class of anticancer immunotherapeutic. Oncolytic viruses preferentially infect and kill cancer cells but not normal cells [4–6], an effect that is largely attributed to the marked genetic differences between cancer cells and normal cells, including defects in innate immune, aberrant oncogenic signaling, and the emergence of tumor-specific receptors [7]. Moreover, oncolytic viruses can kill malignant cells by inducing an inflammatory microenvironment and modulating the tumor vasculature [8, 9]. Given these traits, oncolytic viruses have become promising anticancer agents.

The oncolytic herpes simplex virus 1 (HSV-1) talimogene laherparepvec (T-VEC), which expresses human GM-CSF, was the first oncolytic immunotherapeutic agent to be approved by the U.S. Food and Drug Administration (FDA) [10]. Indeed, the development of oncolytic viruses as antineoplastic drugs has accelerated since this approval was granted, with numerous oncolytic viruses presently in clinical testing [11–13]. However, not all patients show good response to the treatment [14, 15]. As such, there is a need to continue to screen for other novel oncolytic viruses to expand the group of patients that could benefit from oncolytic therapy, with the ultimate goal for developing personalized screening tools for treatment with specific oncolytic viruses.

To identify novel oncolytic viruses with good safety and efficacy, we previously isolated numerous strains of attenuated viruses from 18 live-attenuated veterinary and avian vaccines, all of which have proven safety profiles in animals and humans. Unexpectedly, the attenuated pseudorabies virus (PRV) strain, HB2000, exhibited rapid and strong cytopathic effects in all four of the tested cancer cell lines, with no effects in the normal cell line. PRV, a member of the *Alphaherpesvirinae* subfamily and *Varicellovirus* genus, is the causative agent of pseudorabies (PR) or Aujeszky’s disease [16], a viral disease in domestic and wild animals that infects the nervous system and other organs, including the respiratory tract, resulting in encephalomyelitis and respiratory disease. Although swine are the primary host and reservoir of PRV [17], this virus is also lethal to numerous other species of mammals, except for higher primates and humans [18]. The PRV genome is a double-stranded DNA molecule (~ 143 kb long) with strong molecular stability and encoding at least 72 genes [19, 20], including gE, gI and TK [21]: gE and gI are major virulence factors of PRV [22], whereas TK is associated with virulence and reactivation of PRV infection from latency [23]. The attenuated PRV vaccine strain HB2000, with deletion of gI, gE and TK, has been used to prevent PRV outbreaks worldwide since 2016 [24].

In this study, we sought to verify the oncolytic activity of PRV-LAV HB2000 in vitro and in vivo. We show that PRV-LAV efficiently cleared syngeneic tumors in immunocompetent mice as well as subcutaneous xenografts of several human cancer cell lines in nude mice, significantly prolonging their survival. Furthermore, an immune response was stimulated in cancer cells infected with PRV-LAV, with the local infiltration of lymphocytes. PRV-LAV treatment was also shown to relieve the immune suppression of tumor-infiltrating CD8^+^ T cells. In combination with immune checkpoint inhibitors (ICIs), the therapeutic response was significantly increased. These findings indicate the potential utility of live-attenuated vaccines like PRV-LAV HB2000 as novel oncolytic agents against malignancy.

## Materials and methods

### Viruses

PRV strain HB2000 was isolated from a live-attenuated vaccine and cultured in PK-15 adult pig kidney epithelial cells. PRV-mNG is a recombinant derivative of HB2000 expressing mNeonGreen. Virus titers were determined by PFU assay using PK-15 cells. HSV-1 strain 17 and OVH [25] were grown in U2OS osteosarcoma cells, with virus titers in these cells also determined by PFU assay.

### Antibodies and reagents

Antibodies against the following proteins were used in this study: Bip (3177 s, Cell Signaling Technology [CST]), eIF2α (5324P, CST), Phospho-eIF2α (3398P, CST), SAPK/JNK (9252S, CST), Phospho-SAPK/JNK (9255S, CST), Caspase-12(2202S, CST), Cleaved Caspase-3 (9661S, CST), Cleaved PARP (9541S, CST), Phospho-EGFR and EGFR (11862S, CST), GAPDH (60,004–1-Ig, Proteintech). PE anti-human EGFR Antibody (352,904, Biolegend). The kinase inhibitor library was purchased from Shanghai Topscience Co., Ltd (Shanghai, China). Afatinib (HY-10261) were purchased from MCE.

### Cells

Cell lines were purchased from ATCC and China National Infrastructure of Cell Line Resource. Cell sources was added in Supplementary Materials Table S1. Cells were cultured in DMEM, RPMI-1640, or F-12 supplemented with 10% (vol/vol) FBS and 1% penicillin/streptomycin (Life Technologies). Primary normal cells were purchased from ATCC and cultured according to the instructions provided.

### Immunofluorescence assay

One day prior to immunofluorescence assay, PK-15 cells were seeded onto circular cover glass placed into the wells of 24-well tissue culture plates (NUNC, Rochester, USA). PRV was added and infected MDCK cells. At 0, 6, 12 and 24-h post-PRV infection, the cells were fixed with 4% paraformaldehyde in PBS for 10 min in the dark. Cells were then permeabilized with 0.1% Triton X-100 in PBS (PBST) for 15 min at room temperature (RT) and blocked with goat serum. Cells were then incubated with the appropriate dilutions of anti-H5H8 and anti-PRV gB antibodies at 37 °C for 30 min, then washed five times with PBS. GAM-FITC was added and incubated for 30 min. The assay plates were again washed five times with PBS. Finally, cells were stained with DAPI nuclear staining for 5 min before observation via confocal microscopy (MRC-1024, Bio-Rad, Hercules, CA).

### Cell viability assay

Cells were seeded in 96-well tissue culture plates (NUNC, Rochester, USA) at 10,000 cells per well in 100 μl of cell-appropriate medium. Viruses were added and cultured as described in each section. The percentage of viable cells was then evaluated using a CCK8 (HY-K0301, MCE) assay cells, according to the manufacturer’s instructions. IC50 values were calculated using GraphPad Prism 8.

### High-throughput screening assay

Glioblastoma multiforme (GBM) cells and PK-15 cells were seeded at a density of 20,000 cells per well in 96-well flat-bottom microplates (NUNC, Rochester, USA). On day one of the assay, diluted kinase inhibitors (final concentrations, 5 μmol/L) and 100 PFUs of PRV-mNG were added to the cells for 48 h at 37 °C in 5% CO_2_. Cellular fluorescence images were acquired with an Opera Phenix High Content Screening System (PerkinElmer Inc., USA), and the number of fluorescent cells was analyzed and counted with the associated Harmony imaging and analysis software.

### Western blot analyses

Cells were lysed with RIPA buffer (R0278, Sigma-Aldrich) and subjected to sodium dodecyl sulfate–polyacrylamide gel electrophoresis (SDS–PAGE). Reactive protein bands were visualized in a ChemiDoc MP System (Bio-Rad) using SuperSignal West Femto Maximum Sensitivity Substrate (Pierce).

### Immunoprecipitation

HepG2 cells, infected for 48 h at MOI = 0.01, were lysed in radio-immunoprecipitation assay (RIPA) buffer (20 mM Tris–HCl, pH 7.5, 150 mM NaCl, 2.5 mM MgCl_2_, 5% glycerol and 0.5% Triton X-100) containing 1 mM phenylmethylsulfonyl fluoride (PMSF) and Roche Complete protease inhibitor cocktail (Roche Diagnostics Ltd, Mannheim, Germany). Lysates were centrifuged at 15,000 g for 20 min at 4 °C. Lysates were then precleared with protein A-Sepharose beads (Invitrogen) and centrifuged at 15,000 g for 5 min. Lysates were then incubated with ~ 2 μg NRP1-hFc (10,011-H02H, Sino Biological Inc.) (5 μg/ml) conjugated beads at 4 °C overnight. Beads were washed thrice in PBS to remove unbound proteins, suspended in 2 × SDS-sample buffer, and boiled for 5 min. Protein complexes were analysed by western blotting using specific antibodies for detection.

### Affinity assay

Surface plasmon resonance (SPR) was used to ascertain affinity to PRV gB peptides and NRP1. Briefly, biotin gB peptides were pre-incubated with streptavidin for 2 h at 4 °C. NRP1-hFc protein was linked to a protein A sensor chip (GE Healthcare). Then, serially diluted samples of the peptide-streptavidin complex (200, 100, 50, 25, 12.5, 6.25, 3.125, 1.5625, and 0.78125 nM) was flowed through the sensor surface at a flow rate of 30 μL/min in PBS-P + buffer (0.2 M phosphate buffer with 27 mM KCl, 1.37 M NaCl, and 0.5% Surfactant P20 (Tween 20)). The flow durations were 120 s for the association stage and 200 s for dissociation. Finally, association rates (ka), dissociation rates (kd), and affinity constants (KD) were calculated using evaluation software equipped for the Biacore 8K instrument (GE Healthcare).

### Caspase Activity Detection

For the detection of caspase-3/7 and caspase-9 activities, cells were cultured in 96-well plates, infected with PRV-LAV HB2000 (MOI = 1), and evaluated using Caspase-Glo Assay Systems (Promega) according to the manufacturer’s protocols. Values were normalized to cell viability (CCK8 assay) at each time point, and the data are presented as the percentage of the control.

### EGFR overexpression and knockdown

The PiggyBac Dual Promoter Vector (System Biosciences) was used as a backbone to construct the EGFR overexpression vector. EGFR-overexpressing cell lines were constructed by transfection of the EGFR overexpression vector and selection with 1 μg/ml puromycin. The expression of EGFR was knocked down by lentivirus-shRNA-EGFR (sc-29301-V) purchased from Santa Cruz Biotechnology.

### Tumor-infiltrating lymphocyte (TIL) isolation and flow cytometric analysis

Mice received four doses of PRV-LAV. Eight days after the final dose, mice were sacrificed and tumors were harvested to isolate and analyze TILs. Tumor weights were recorded, and then the tumors were minced with scissors and incubated with 1 mg/ml collagenase D (11088866001, Roche) and 0.5 mg/ml DNase I (11284932001, Sigma-Aldrich) in RPMI-1640 medium supplemented with 2% FBS at 37℃ for 1.5 h with continuous agitation. The digestion mixture was homogenized by repeated pipetting and filtered through a 70-μm nylon filter. The cell suspension was centrifuged at 50 × g for 10 min at 4℃. The supernatant was collected and centrifuged at 300 × g for 5 min at 4℃. The cell pellets were washed twice with PBS at 300 × g for 5 min at 4℃ and suspended in 2 ml of ACK lysis buffer (R1010, Solarbio) for 1 min to deplete red blood cells. The cell pellets were resuspended in 3 ml of 40% Percoll (P7828, SIGMA-ALDRICH) and slowly added to tubes containing 3 ml of 80% Percoll. The samples were centrifuged at 1,625 × g for 60 min at room temperature. Cells at the interface between the 40% and 80% Percoll were collected. After two washes, the cells were stained with the corresponding antibodies and incubated for 30 min at 4 °C for flow cytometric analysis in a BD LSRFortessa X-20. Data were analyzed with FlowJo.

The antibodies used for flow cytometry were: anti-mouse CD4-APC/Cy7 (100414); anti-mouse NK1.1 PerCP-Cy5.5 (108728); anti-mouse CD8α-PE/Cy7 (100722); anti-mouse CD11b PE (101208); anti-mouse B220-BV421 (103251); anti-mouse CD80 FITC (104706); anti-mouse CD86-BV421 (105031); anti-mouse Ly6c-APC/Cy7 (128026); anti-mouse Ly6g-BV605 (127639); anti-mouse CD279-BV421 (135218); anti-mouse CD223-APC (125210); anti-mouse CD152-PE (106306); anti-mouse CD366-BV605 (119721), all from Biolegend. Anti-mouse CD11c-PerCP/Cy5.5 (560584) was from BD, and LIVE/DEAD Fixable Aqua Dead Cell Stain Kit was from Molecular Probes (L34966).

### Cell profiling using CyTOF

Single-cell suspensions of TILs were prepared as described previously. For the CyTOF assay, unconjugated antibodies (Supplementary Materials Table S2) were obtained from Fluidigm and conjugated in-house using the Maxpar R X8 Multimetal Labeling Kit (Fluidigm), according to the manufacturer’s instructions. Briefly, single-cell suspensions were stained with 1 μM cisplatin (Fluidigm) for 15 min and then blocked with Fc receptor blocking buffer (Biolegend) for 10 min at room temperature. Cells from each sample were incubated with a metal-conjugated surface antibody cocktail on ice for 30 min and barcoded with a unique combination of palladium metal barcodes according to the manufacturer’s instructions (Fluidigm). Next, the cells were pooled together, and then fixed and permeabilized using the Nuclear Staining Buffer Set (Fluidigm). The cells were subsequently stained with a metal-conjugated intracellular antibody cocktail for 30 min at 4 °C. After washing, the cells were incubated in 1 ml of intercalator buffer (0.125 nM MaxPar Intercalator-Ir in 1 ml of Fixation & Permeabilization Buffer).

Prior to acquisition, the cells were diluted to 8 × 10^5^ cells/ml in deionized water containing 10% EQ Four Element Calibration Beads (Fluidigm) and filtered through a 70-μm nylon filter. Events were acquired on a CyTOF 2 Helios upgraded mass cytometer at an event rate of 200–300 cells/second at the Flow Cytometry and Cellular Imaging Facility of Xiamen University. The ‘.fcs’ files were normalized to the EQ 4-element bead signal (Lot P15K0802, Passport EQ 4_P13H2302) in 100-s interval windows using normalization software (version 6.7.1014, Fluidigm). Mass tag barcodes were resolved with a doublet filtering scheme using Debarcoder (Fluidigm). Live immune cells were manually gated with FlowJo by event length, live/dead discrimination, and the expression status of CD45. Data were then exported for downstream analysis and transformed with a coefficient of 5 with the cytofAsinh method. For downstream analyses, individual sample data were subsampled to 10,000 events of the CD45^+^ population. Contour plots were used to specifically define the T cell clusters in manual gates with FlowJo, and were exported as ‘.fcs’ files. t-SNE dimensionality reduction and PhenoGraph clustering analyses were performed using the tool cytofkit run in R package software. Partial markers were used during the t-SNE and PhenoGraph analyses. For the generation of heatmap displays, marker expression was normalized by dividing by the range of all markers (expression range from the 1st to 99th percentile). Data displays were generated using the ggplot2 R package.

### Animal experiments

Complete response (CR) means the tumor has been cleared during the observation period. Partial response (PR) means tumor growth has been inhibited and less than 70% of the maximum tumor volume (2000 mm^3^) during the observation period.

For the subcutaneous xenograft model, GBM (5 × 10^6^ cells/mouse), HepG2 (5 × 10^6^ cells/mouse), A549 (3 × 10^6^ cells/mouse) cells and human liver cancer tissues (LIHC 0184006) were inoculated subcutaneously into the hind-flanks of 6-week-old female BALB/c nude mice and NOD-SCID mice. Tumors developed to ~ 100 mm^3^ sizes, and mice were randomly divided into two groups. Mice were administered with four intralesional injections of PRV (1 × 10^7^ PFUs/dose, 50 µl) or control (DMEM, 50 µl) on the 1st, 3rd, 5th, and 7th days. Tumor sizes were measured daily, converted to tumor volumes and plotted as tumor growth curves. For survival experiments, mice were monitored for tumor-growth and euthanized before tumors reached 2000 mm^3^. Kaplan–Meier curves were used to calculate survival.

To establish syngeneic mouse models, Hepa1-6 (5 × 10^6^ cells/mouse) and CT26 (2 × 10^6^ cells/mouse) cells were inoculated subcutaneously into the hind-flanks of 6-week-old female BALB/c or C57BL/6 mice. Tumor lesions were allowed to grow to a volume of ~ 100 mm^3^. Mice were then administered with four intralesional injections of PRV-LAV (1 × 10^7^ PFUs/dose, 50 µl) or control (DMEM, 50 µl) on the 1st, 3rd, 5th, and 7th days. The tumor sizes in the two groups were measured daily, converted to tumor volumes and plotted as tumor growth curves. For survival experiments mice were monitored for tumor-growth and euthanized before tumors reached 2000 mm^3^. Kaplan–Meier curves were used to calculate survival.

For re-challenge experiments, Hepa1-6 (5 × 10^6^ cells/mouse) and CT26 (2 × 10^6^ cells/mouse) cells were inoculated subcutaneously into the single hind-flank of 6-week-old female BALB/c or C57BL/6 mice. Tumor lesions were allowed to grow to a volume of ~ 100 mm^3^. Mice were then administered with four intralesional injections of PRV-LAV (1 × 10^7^ PFUs/dose, 50 µl) or control (DMEM, 50 µl) on the 1st, 3rd, 5th, and 7th days. Cured mice from PRV-LAV therapy were rechallenged on day 60 with a two-fold increased number of the same cancer cells in the contralateral (left) flank. Age matched (14 to 16-week-old) naïve mice were used as controls (*n* = 6).

For double combination studies using PRV-LAV + anti-checkpoint antibodies, BALB/c mice were implanted subcutaneously in the right flank with 2 $\times$ 10^6^ CT26 cells. Tumor sizes developed (~ 100 mm^3^), and then mice were randomly divided into eight groups (*n *= 6/group). Mice were then treated with PRV-LAV (5 × 10^6^ PFUs/dose, 50 µl) or control (DMEM, 50 µl) via intratumoral injections on days 1, 3, 5, 7, or αPD-1 antibody (5 mg/kg, clone: 29F.1A12, Bioxcell), αCTLA4 antibody (5 mg/kg, clone: UC10-4F10-11, Leinco) or isotype control antibody via intra-peritoneal (i.p) injection on days 2, 4, and 6.

Depleting anti-CD8 antibodies (clone 53.6.72), anti-CD4 antibodies (clone GK1.5), and isotype control antibodies were obtained from BioXCell. 400 μg depleting antibodies or rat IgG isotype control antibodies were intraperitoneally injected 2 days before virus treatment, and then repeatedly injected on days 0, 2 and 4 after injection. 10^7^ PFU of virus was intratumorally injected when the tumors reached 100 mm^3^, and then repeatedly injected on days 2, 4, 6 after the initial treatment. Tumor size was monitored for 27 days.

### Hematology

Whole blood collected in EDTA-coated tubes was used to analyze complete blood counts and differential counts in an automated hematology analyzer (BC-5300VET) according to the manufacturer’s instructions.

### Statistics

Statistical parameters and methods are reported in the Figures and the Figure Legends. Survival data were analyzed by the log-rank test. For all statistical analyses, differences were considered significant when the *P* value was less than or equal to 0.05. (*P < 0.05; **P < 0.01; ***P < 0.001; ****P < 0.0001). Statistical analyses were performed using GraphPad Prism 8.

## Results

### Oncolytic activity of HB2000 isolated from live-attenuated vaccines

**Fig. 1:**
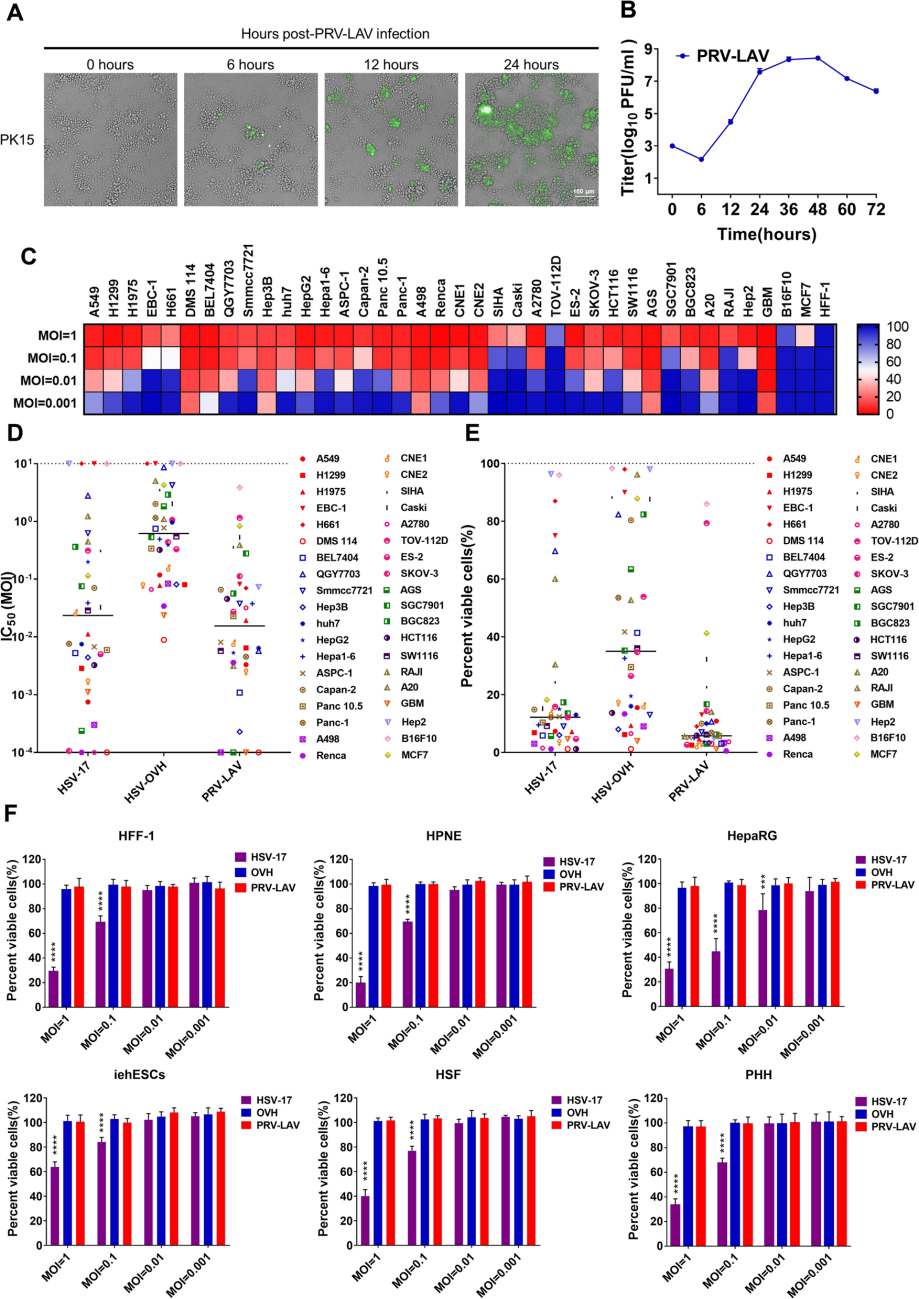
Selective oncolytic efficacy of PRV-LAV in vitro. **A** PK-15 cells infected with the PRV-LAV HB2000 strain (MOI = 0.001) were specifically labeled with an antibody against PRV gB and then imaged via phase-contrast and fluorescence microscopy. Green staining, infected cells positive for gB protein. Scale bar, 100 μm. **B** PK-15 cells were infected with PRV-LAV. Single-step growth analyses were conducted. Virus was collected from both the supernatant and cell lysate. **C** Cell viability assays were performed in 38 representative cancer cell lines at 72 h after exposure to PRV-LAV HB2000. The results are visualized on a heat map generated with GraphPad Prism 7. The values are the average of three independent experiments. **D**, **E** The half-maximal inhibitory concentration (IC_50_) values (**D**) and percentage of viable cells (MOI = 1) (**E**) in 38 cancer cell lines treated with HSV-17, OVH and PRV-LAV HB2000 were determined from three independent experiments. Each value is plotted as a single symbol. The IC_50_ values were determined by nonlinear regression fitting using GraphPad Prism software. Bars indicate the mean values. Cancer cell lines were selected to cover the major types of cancer. Red, lung cancer; blue, liver cancer; brown, pancreatic cancer; violet, renal cell carcinoma; light orange, nasopharyngeal carcinoma; black, cervical carcinoma; pink, ovarian cancer; green, gastric cancer; deep purple, colon cancer; light brown, lymphoma; orange, glioma; light blue, laryngeal cancer; light pink, melanoma; light yellow, mammary cancer. **F** Cell viability assays were performed on normal human cells at 72 h after exposure to HSV-1 strain 17, OVH and PRV-LAV HB2000. The black bars indicate the mean values. One-way ANOVA was used to determine the significance of differences in the percentages of viable cells after infection with PRV-LAV and compared with those in the groups treated with HSV-17 and OVH. *P < 0.05, **P < 0.01, ***P < 0.001, ****P < 0.0001. Human foreskin fibroblasts, HFF-1; human pancreatic ductal epithelial cells, HPNE; human hepatocytes, HepRG; human uterine stromal cells, iehESCs; primary skin fibroblasts, HSFs; primary human hepatocytes, PHHs

To determine appropriate viral culture conditions, we first sought to culture the PRV-LAV HB2000 strain in adult porcine kidney epithelial PK-15 cells. We used immunofluorescence and an antibody specific for glycoprotein B (gB) of PRV to detect virus expression in PK-15 cells at 0, 6, 12, and 24 h after PRV-LAV infection. Time-lapse microscopy showed that the isolated HB2000 strain replicated rapidly in PK-15 cells (Fig. 1A). Through kinetics evaluation of PRV-LAV HB2000 in PK-15 cells in vitro (Fig. 1B), we identified peak virus titers at 48 h.

Next, we determined the tumoricidal activity of PRV-LAV in 38 cultured human and mouse cancer cell lines. Of note, PRV-LAV efficiently induced cell death in most cancer cells by 72 h after infection (Fig. 1C). We noted particularly efficient and broad activity against lung cancer, hepatocellular carcinoma, pancreatic carcinoma, renal carcinoma, and nasopharyngeal carcinoma cells as compared with other types of cancer cell lines. Thus, oncolytic PRV-LAV may have diverse applications as an immunotherapeutic agent.

HSV-1 T-VEC is the only oncolytic immunotherapeutic with FDA approval to date. Given that PRV and HSV-1 belong to the *Alphaherpesvirinae* subfamily, we next sought to determine whether PRV and HSV-1 shared oncolytic characteristics. To this end, we compared the half-maximal inhibitory concentration (IC_50_) values of the HSV strain 17 (HSV-17, a wild-type strain of HSV-1), the oncolytic HSV-1 OVH (OVH, an engineered modified HSV-1 KOS strain deleted ICP0 and ICP34.5) [25] and PRV-LAV against 38 cancer cell lines as a measure of the tumoricidal activity. We found that the tumoricidal activity of HB2000 was approximately equal to that of HSV-17 and significantly higher than that of OVH (Fig. 1D). In addition, the percentage of viable cells after PRV-LAV infection was approximately equal to that after HSV-17 infection and significantly greater than that after OVH infection (Fig. 1E). Interestingly, in some of the cancer cell lines, such as Hep-2, QGY7703, and Raji, almost no cell death was observed after infection with HSV-17 at high MOIs; yet, all three cell lines were readily killed by PRV-LAV at low MOIs. Conversely, PRV-LAV at high MOIs had minimal effect in other cell lines, such as TOV-112D and MCF7, cell lines that were readily killed by HSV-17 at low MOIs. Thus, the tumoricidal activity of PRV-LAV is distinct and cell-type specific.

Next, we examined the effect of the three viruses on the viability of normal human cells (human foreskin fibroblasts [HFF-1], human pancreatic ductal epithelial cells [HPNE], human hepatocytes [HepRG], human uterine stromal cells [iehESCs], Human skin cell fibroblasts [HSFs], and primary human hepatocytes [PHHs]) (Fig. 1F). HSV-17 exhibited obvious cytopathic effects in normal cells at 72 h after infection at multiple MOIs. In comparison, PRV-LAV and OVH were sufficiently safe, even after infection with up to 1 PFU of virus per cell for 72 h.

To objectively quantify cell death resulting from viral infection, the percent of viable cells was detected using CCK8 assay. The viability of normal human cells infected with HSV-17 was significantly reduced. However, the viability of cells infected with PRV-LAV and OVH was only minimally reduced. Thus, the cancer-selective cytotoxic activity leads to the high tumor tropism of PRV-LAV.

### EGFR-overexpressing cancer cells promote PRV proliferation

**Fig. 2:**
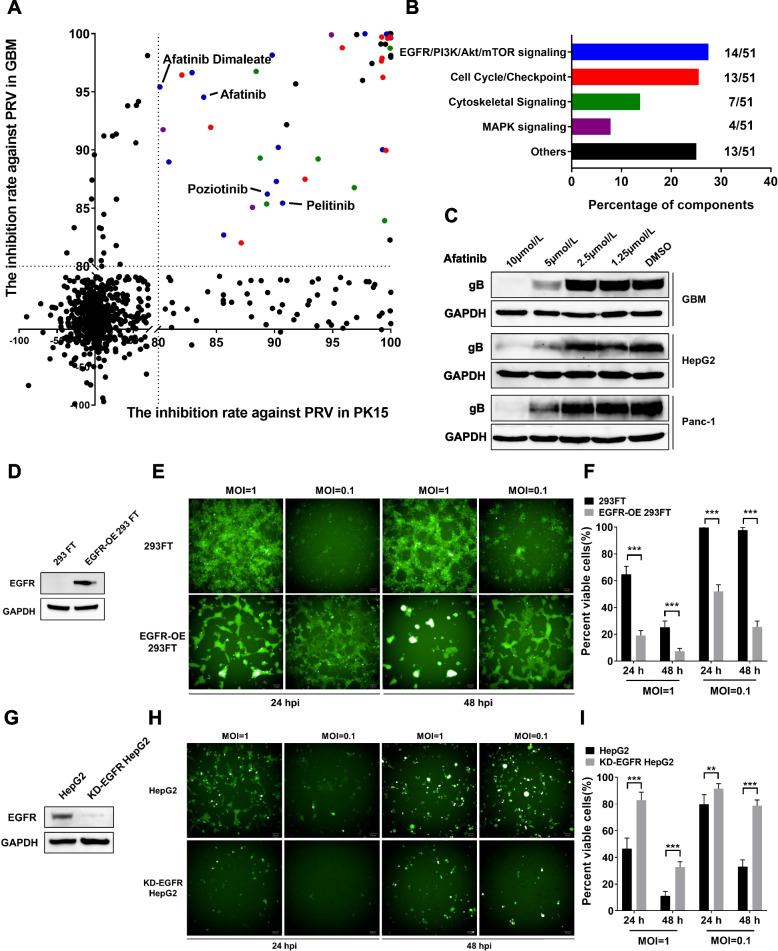
The expression of EGFR regulates the proliferation of PRV-LAV. **A**, **B** GBM cells and PK-15 cells were pretreated with vehicle or 5 μM kinase inhibitor and were then infected with PRV-LAV-mNeonGreen (MOI = 0.001). The inhibition rates against PRV infection in GBM and PK-15 cells were calculated by Harmony imaging and analysis software. The results are presented on a scatter plot. Each point on the plot represents a kinase inhibitor (**A**). Kinase inhibitors with inhibition rates of at least 80% in both GBM and PK-15 cells were classified according to their molecular function in signaling pathways (**B**). This experiment was repeated three times. **C** The expression of PRV gB was analyzed by western blotting after cancer cells were pretreated with the EGFR inhibitor afatinib and then infected with PRV-LAV HB2000 (GBM, MOI = 0.001; HepG2 and Panc-1, MOI = 0.01) and cultured for 48 h. **D** The expression of EGFR in 293FT and EGFR-OE 293FT cells was analyzed by western blotting. **E**,** F** 293FT and EGFR-OE 293FT cells were infected with PRV-LAV-mNeonGreen (MOI = 0.1, 1). Phase-contrast and fluorescence micrographs were acquired with an Opera Phenix High Content Screening System (**E**), with cell viability assays were performed (**F**) 24 h and 48 h post-infection. **E** Scale bars, 100 μm. **F** Data are presented as the mean ± s.d. values (*n* = 6). 293FT cells vs. EGFR-OE 293FT cells. A *t* test was used to determine the significance of differences in the percentages of viable cells post-viral infection. **G-I** Knockdown of EGFR expression in HepG2 cells suppressed the proliferation of PRV-LAV. The expression of EGFR in HepG2 and KD-EGFR HepG2 cells was analyzed by western blotting (**G**). HepG2 and KD-EGFR HepG2 cells were infected with PRV-LAV-mNeonGreen (MOI = 0.1, 1). Phase-contrast and fluorescence micrographs were acquired with an Opera Phenix High Content Screening System (**H**), with cell viability assays were performed (**I**) 24 h and 48 h post-infection. Scale bars, 100 μm. The data are presented as the mean ± s.d. values. Data are presented as the mean ± s.d. values (*n* = 6). The black bars indicate the mean values. A *t* test was used to determine the significance of differences in the percentages of viable cells post-viral infection

The finding that PRV preferentially replicates in and kills cancer cells prompted us to investigate the molecular mechanism of PRV tropism. Protein kinases are known to play key roles in signaling pathways, and their aberrant activation in eukaryotic cells is frequently linked with tumorigenesis. Thus, identifying the protein kinases associated with PRV infection and proliferation may provide insight into its tumor tropism. A kinase inhibitor library was applied to screen for kinase inhibitors that could suppress the proliferation of PRV in the ultrasensitive GBM cells and PK-15 cells. The inhibition rates of the kinase inhibitors against PRV in GBM and PK-15 cells were determined (Fig. 2A), and those that showed inhibition rates above 80% were selected (Table S1), with classifications created according to the signaling pathway of their targeted kinase (Fig. 2B).

Of the various signaling pathways tested, the EGFR/PI3K/Akt/mTOR signaling pathway, cell cycle/checkpoint signaling pathway, cytoskeletal signaling pathway and MAPK signaling pathway appeared to be linked with PRV proliferation. Further, the cell cycle/checkpoint signaling pathway, cytoskeletal signaling pathway, and MAPK signaling pathway demonstrated likely crosstalk with the EGFR/PI3K/Akt/mTOR signaling pathway. Thus, we considered the EGFR/PI3K/Akt/mTOR signaling pathway to be strongly associated with the proliferation of PRV.

In the EGFR/PI3K/Akt/mTOR signaling pathway, EGFR is the gene farthest upstream, and we tested and showed that EGFR inhibitors could strongly suppress the replication of PRV (Fig. 2A). To verify this, we measured the inhibitory activity of the EGFR inhibitor afatinib against PRV proliferation in GBM, HepG2, and Panc-1 cells. Treatment with afatinib effectively suppressed PRV proliferation, with cytopathic effects note (Fig. S1A). Furthermore, tumor cells treated with afatinib had lower viral protein expression levels (Fig. 2C) and fewer viral particles in the supernatant than did untreated cells (Fig. S1B). These results confirmed that inhibition of EGFR can significantly suppress PRV replication.

To verify the influence of EGFR overexpression on PRV replication, we generated 293FT cell lines with stable expression of EGFR (EGFR-OE 293FT) (Fig. 2D), and then infected EGFR-OE 293FT cells and control 293FT cells with PRV at MOIs of 0.1 and 1. After 24 h, PRV replication was faster in EGFR-OE 293FT cells than in the control cells, with stronger oncolytic activity. By 48 h, this phenomenon was enhanced (Fig. 2E). EGFR overexpression also resulted in increased viral replication and cell death (Fig. 2F). This process may be associated with the fusion of PRV-infected EGFR-OE 293FT cells to form multinucleated syncytia, as membrane fusion has previously been reported to facilitate the spread of HSV-1 strains. Finally, we showed that knockdown of EGFR expression in PRV-sensitive HepG2 cells successfully suppressed PRV proliferation (Fig. 2G-I).

EGFR signaling is important for PRV infection and proliferation, and we showed the entry of PRV into cells is accompanied by altered EGFR expression on the cell membrane (Fig. S1C); this may suggest that EGFR participates in PRV entry into cells. However, whether PRV directly interacts with EGFR is unknown.

EGFR stimulation leads to autophosphorylation of the kinase domain of the protein. HepG2 cells were infected with PRV at an MOI of 10 for 5 min, 30 min, 1 h and 2 h to assess for changes in EGFR phosphorylation (pEGFR) by immunoblotting. We found a significant increase in pEGFR at 5 min post-PRV infection that peaked at 1 h post-infection (Fig. S1D). Thus, PRV infection activates EGFR signaling to enhance PRV infection and replication. We identified that the glycoproteins of PRV—gB, gC, gD—as well as the viral particles, were used to activate EGFR signaling, and only gB protein and viral particles can effectively activate EGFR signaling (Fig. S1E). The gB and EGFR colocalization was confirmed via fluorescence microscopy (Fig. S1F). Thus, we suggest that gB can directly activate EGFR signaling at the early stage of infection.

EGFR mediates endocytosis via different pathways. We next sought to determine whether EGFR is linked with PRV-LAV cell entry. HepG2 cells were pre-incubated with inhibitors of macropinocytosis (5-(N-ethyl-N-isopropyl)-amiloride, EIPA), lipid raft-dependent endocytosis (Methyl-β-cyclodextrin, MβCD), or clathrin-mediated endocytosis (Chlorpromazine, CPZ) at the indicated concentrations for 30 min, and then infected with PRV-LAV for 2 h. The cells were then washed with Hanks solution and cultured for 72 h. We found that PRV-LAV infection was suppressed by MβCD and EIPA, but not by CPZ, with cytopathic effects and percent viable cells (Fig. S1G-H). These data demonstrate that PRV-LAV enters cancer cells via macropinocytosis and lipid raft-dependent endocytosis, but not clathrin-mediated endocytosis.

Finally, we sought to confirm a direct interaction between gB and EGFR. However, such binding was unable to be confirmed in vitro. We speculated that another factor is necessary for the interaction between gB and EGFR.

PRV gB has the same furin splice site (RRAR) as the SARS-CoV-2 spike protein, which has been identified as being the basis for SARS-CoV-2 spike protein recognition of NRP1 for virus entry. We hypothesized that PRV gB may interact with NRP1 in our system. The PRV gB CendR peptide (GVVGPASPAAARRAR) has a high affinity for NRP1, and mutation of PRV gB CendR peptide (GVVGPASPAAARRAA) can abolish this binding activity (Fig. S2A-C). Using NRP1 protein to perform immunoprecipitation experiments, we identified both PRV gB and EGFR were both specifically pulled down by NRP1 (Fig. S2D). Through these findings, we suggest that PRV interacts with and activates EGFR via NRP1.

### PRV-LAV induces cancer cell death via endoplasmic reticulum stress

**Fig. 3:**
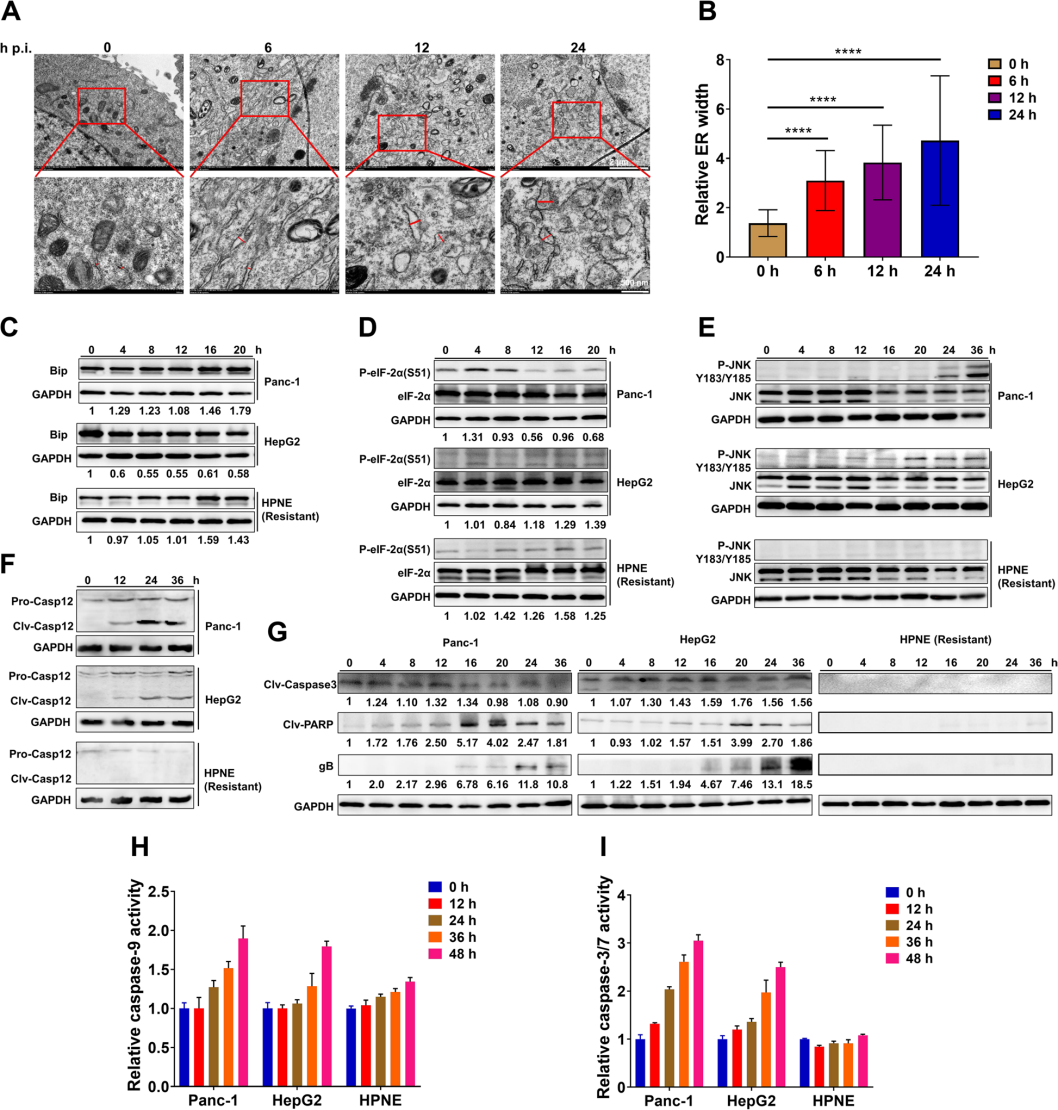
PRV specifically triggers severe ER stress to induce apoptosis in cancer cells. **A** Observation of ER swelling/distension in Panc-1 cells infected with PRV-LAV HB2000 by TEM. High-magnification images are also presented. Scale bars, 500 nm. **B** Quantification of ER distension in (**A**). Data are presented as the mean ± s.d. values (*n* = 40). Statistical analysis was performed by one-way ANOVA. **C-G** Effect of PRV-LAV on the ER stress-induced apoptosis signaling. Western blot analysis of Bip (**C**), phosphorylated eIF-2α and eIF-2α (**D**), phosphorylated JNK and JNK (**E**), caspase-12 (**F**), cleaved caspase-3, cleaved PARP, and PRV gB (**G**). GAPDH was used as a loading control. **H**-**I** Caspase-9 (**H**) and caspase-3/7 (**I**) activity in Panc-1, HepG2, and HPNE cells treated with PRV-LAV HB2000 (MOI = 1). Data are presented as the mean ± s.d. values (*n* = 3). TEM, transmission electron microscopy

Tendentiously, we surmised that PRV-LAV infects EGFR-overexpressing cancer cells. However, the mechanism by which PRV-LAV induces cancer cell death is unknown. Therefore, we next explored the effect of virus infection on host cell morphology using transmission electron microscopy (TEM). Swelling/enlargement of the lumen of the endoplasmic reticulum (ER) was observed as early as 6 h post-PRV-LAV infection in Panc-1 cells (Fig. 3A), which is direct morphological evidence of ER stress. Furthermore, we noted progressive distension of the ER over time (Fig. 3B), suggesting that PRV-LAV might induce cancer cell apoptosis via severe ER stress.

The main cause of the ER stress response is abnormal protein synthesis and its accumulation in the ER. Thus, we next assessed the expression levels of GRP78 (Bip), a well-known marker of ER stress, in PRV-sensitive Panc-1 and HepG2 cancer cells, and in PRV-resistant HPNE normal cells post PRV-LAV infection. By western blotting, we noted an increase in the expression of Bip after PRV-LAV infection in Panc-1 and HPNE cells (Fig. 3C). EIF2α is a translation initiation factor that is phosphorylated by protein kinase RNA-like endoplasmic reticulum kinase (PERK) in response to ER stress. Phosphorylation of eIF-2α globally suppresses protein translation to reduce ER stress [26]. We noted phosphorylation of eIF-2α expression to be obviously increased in Panc-1, HepG2 and HPNE cells post PRV-LAV infection, with clear decreased expression in Panc-1 cells (Fig. 3D).

We next examined the signaling pathways associated with ER stress-induced apoptosis. Western blot analyses revealed that the c-Jun N-terminal kinase (JNK) and caspase-12 pathways were strongly induced in PRV sensitive Panc-1 and HepG2 cells after PRV-LAV infection (Fig. 3E-F), but no change in HPNE cells (Fig. 3E-F). We also evaluated the apoptotic markers caspase-3 and PARP and found both to be cleaved in Panc-1 and HepG2 cells after PRV-LAV infection; albeit these signals were difficult to detect in HPNE normal cells (Fig. 3G). PRV glycoprotein gB is specifically expressed in Panc-1 and HepG2 cells, suggesting that the occurrence of apoptosis in these tumor cells is related to virus replication (Fig. 3G). Finally, HepG2 and Panc-1 cells had higher caspase 9 and 3/7 activity (Fig. 3H-I). Thus, we can conclude that PRV-LAV induces cancer cell apoptosis via ER stress.

### PRV-LAV safety in experimental animal models

PRV-LAV has strong tumor killing activity. Therefore, we sought to determine the therapeutic effect of PRV-LAV on tumors in vivo. To this end, we first established the safety profile of PRV-LAV in mice and rats after PRV-LAV treatment. We intravenously (i.v.) injected five doses of PRV-LAV (1 × 10^8^ PFUs/dose) into immunocompetent BALB/c mice and Sprague–Dawley (SD) rats; the dosing schedule is shown in Fig. S3A. Injections were performed every other day for a total of five injections. Body weight was measured daily, and routine blood tests and blood biochemical analyses were performed every three days.

Overall, we found that none of the PRV-LAV- or mock-injected animals displayed obvious disease symptoms over the experimental period, with no significant changes in body weight for any of the animals (Fig. S3B-C). Hematological studies revealed no significant change in white blood cell, platelet, or red blood cell (RBC) counts between the PRV-LAV- and mock-injected groups (Fig. S3D-I). Liver aspartate aminotransferase (AST) and alanine aminotransferase (ALT) levels in the PRV-LAV- and mock-injected mice and rats remained unchanged (Fig. S3J-M). Blood urea nitrogen concentration, as a marker of kidney function, was also unchanged (Fig. S3N-O). Overall, PRV-LAV has an excellent safety profile in vivo with the potential to be used to treat malignant tumors in humans.

### PRV-LAV exhibits effective antitumor therapeutic activity in vivo

**Fig. 4:**
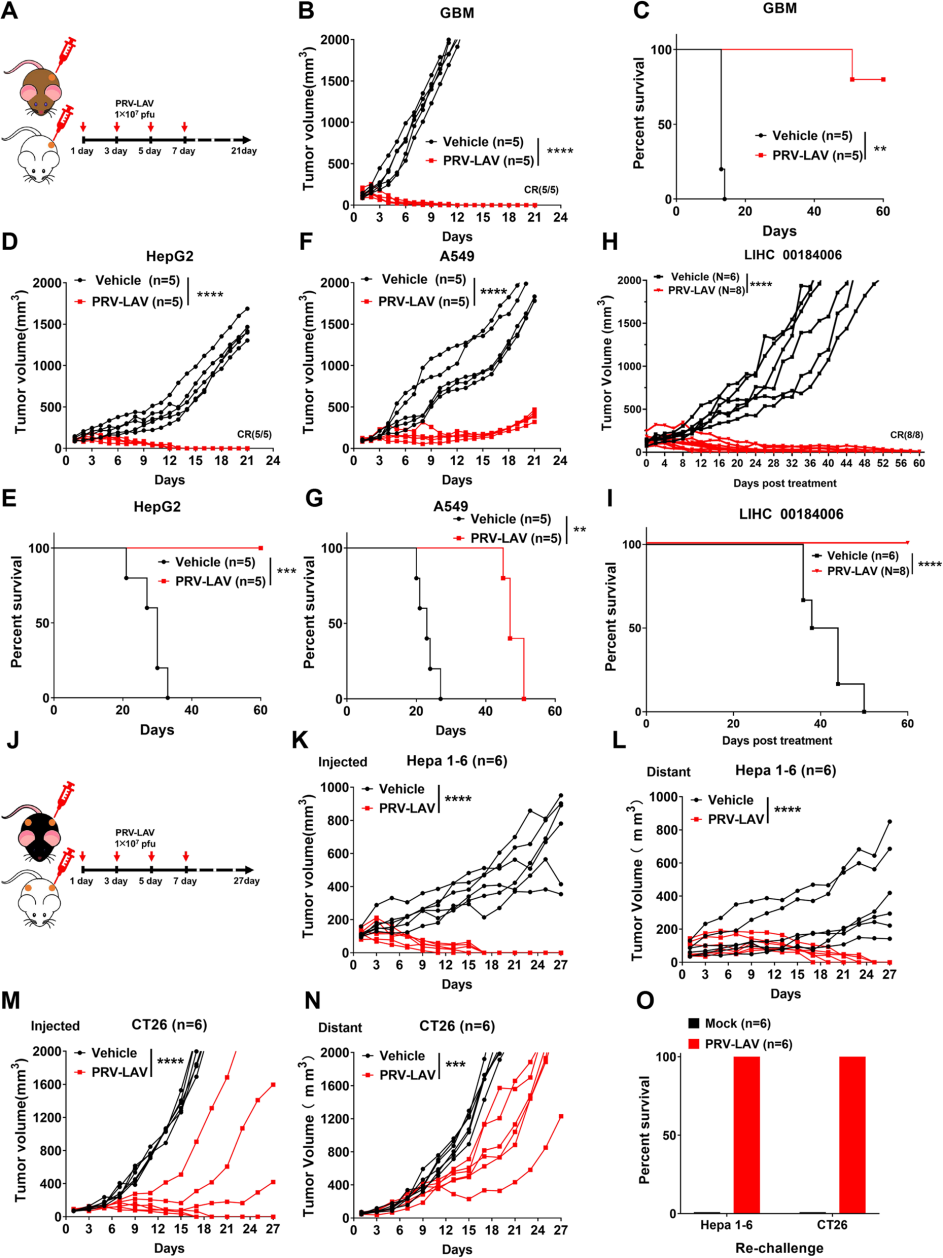
In vivo therapeutic efficacy of PRV-LAV. **A** Timeline of the experimental setup for the experiments in the Balb/c nude or NOD-scid mouse model. **B-G** Tumor volume curves (**B**, **D**, **F**) and Kaplan–Meier survival curves (**C**, **E**, **G**) for mice bearing GBM, HepG2, and A549 tumors treated with vehicle or PRV-LAV (1 × 10^7^ PFUs, intratumorally). **H-I** Therapeutic activity of PRV-LAV in the liver cancer PDX model (LIHC 00184006). Tumor volume curves (**H**) and Kaplan–Meier survival curves (**I**) for PDX mice treated with 4 doses of vehicle (*n* = 6) or PRV-LAV (*n* = 8) (1 × 10^7^ PFUs, intratumorally). **J** Timeline of the experimental setup for the experiments in Hepa1-6 and CT26 syngeneic models in immunocompetent mice. **K-N** Changes in the injected (**K**, **M**) and distant (**L**, **N**) tumor volumes curves for mice bearing Hepa1-6 and CT26 tumors treated with vehicle or PRV-LAV (1 × 10^7^ PFUs, intratumorally). In (**B**, **D**, **F**, **H**, **K**-**N**), comparisons were performed by AUC analysis. Statistical analysis was performed by *t* test. *P < 0.05; **P < 0.01; ***P < 0.001; ****P < 0.0001. Statistical analysis was performed using the log-rank test in (**C**, **E**, **G**, **I**). **O** Tumor cells were inoculated subcutaneously into the single hind-flank of mice. After 60 days post PRV-LAV treatment, cured mice treated with PRV-LAV were rechallenged with two-fold increased number of the same cancer cells. Recurrence rates were monitored in all groups

To explore the therapeutic efficacy of PRV-LAV, cancer xenograft mouse models were established through the subcutaneous injection of glioblastoma multiforme (GBM) cells, HepG2 cells, or A549 cells into female BALB/c-nude mice. Tumor lesions were grown to a volume of ~ 100 mm^3^ and mice were then treated with PRV-LAV or a control. We found that tumors established with HepG2 and GBM cells in nude mice were completely cleared following treatment with PRV-LAV, and showed a lack of recurrence of an extended timeframe (Fig. 4A-E). Similarly, tumors formed from A549 cells in nude mice were also obviously inhibited by treatment with PRV-LAV (Fig. 4F-G).

The PDX model is based on the transfer of primary tumors directly from the human patient into an immunodeficient mouse. Here, we used the human liver hepatocellular carcinoma (LIHC 00184006) patient-derived xenograft (PDX) model to evaluate the therapeutic activity of PRV-LAV. We found that tumors in the PDX mouse were cleared and showed no recurrence after PRV-LAV treatment (Fig. 4H-I). Thus, PRV-LAV provides effective antitumor therapeutic activity in immunodeficient mouse models.

To explore the therapeutic activity of PRV-LAV in immunocompetent mice, syngeneic mouse models were established using Hepa1-6 and CT26 cells. Like in the xenograft mouse model, we found that injected and distant tumors formed from Hepa1-6 cells were completely cleared and did not recur for a prolonged period after PRV treatment (Fig. 4J-L). The growth of tumors from CT26 cells were significantly inhibited after PRV treatment as compared with that of control tumors, with injected tumors completely cleared in 50% of mice and distant tumors not cleared (Fig. 4M-N). PRV-LAV treatment was able to induce antitumor immune memory and 100% resistance to a second challenge with tumor cells (Fig. 4O). These results show that PRV-LAV possesses effective antitumor therapeutic activity in a syngeneic model in immunocompetent mice.

### PRV-LAV exerts antitumor activity by activating the anti-tumor function of CD8^+^ T cells

**Fig. 5:**
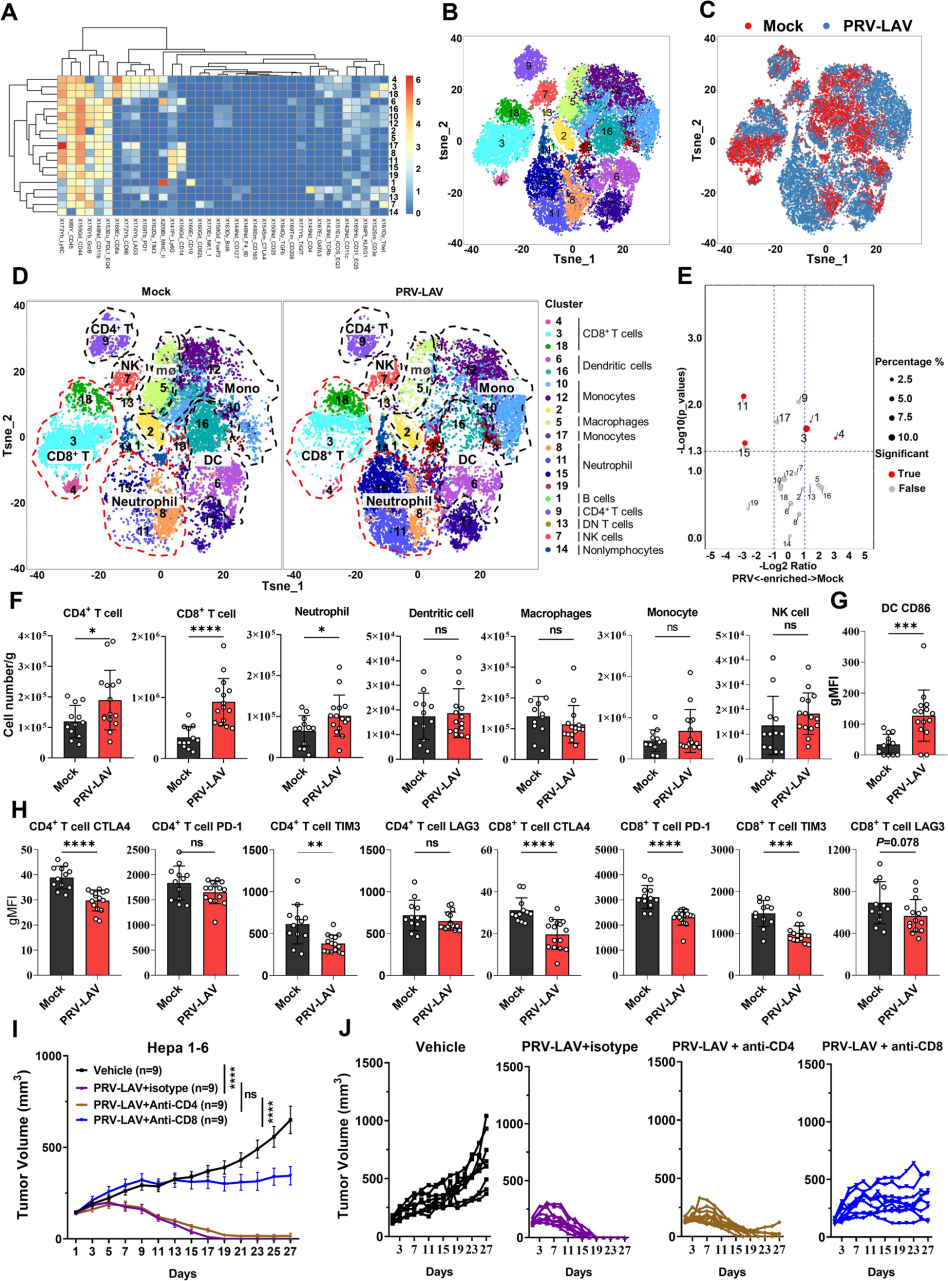
PRV-LAV treatment effectively relieves the immunosuppressive state of T cells in a Hepa1-6 mouse model. **A** Heatmap displaying normalized marker expression of each CD45^+^ cell cluster. **B** t-SNE plot derived from CyTOF analysis of tumor immune infiltrates obtained from each treatment group. Cells are colored by the clusters identified by PhenoGraph. **C-D** Density t-SNE plots of equal numbers of tumor immune infiltrates from vehicle and PRV-LAV treatment group. Merged (**C**) and separate (**D**) images are shown. Mø, macrophage; DC, dendritic cell; Mono, monocytes. **E** Quantitative analysis of each cell cluster as a percentage of CD45^+^ cells. *P* values were calculated by paired* t* test. Cluster 1: B cells; cluster 4: exhausted CD8^+^ T cells; cluster 3: activated CD8^+^ T cells; cluster15: naïve neutrophils; cluster11: activated neutrophils. **F–H** Tumor immune infiltrates were analyzed by flow cytometry the day after mice received the final dose of 4 doses of mock (*n* = 12) or PRV-LAV (*n* = 15) treatment. The numbers of tumor-infiltrating CD4^+^ T cells, CD8^+^ T cells, neutrophils, dendritic cells, macrophages, monocytes, and NK cells per gram tumor were calculated (**F**). The mean fluorescence intensity of CD86 on DC cells (**G**), CTLA4, PD-1, TIM3, and LAG3 on CD4^+^ and CD8^+^ T cells (**H**) were counted. The data are presented as the mean ± s.d. values. The black bars indicate the mean values. Statistical analysis was performed by *t* test. *P < 0.05; **P < 0.01; ***P < 0.001; ****P < 0.0001. **I-J** The knockout of CD4^+^ and CD8^+^ T cells during PRV-LAV treatment. Changes in the tumor volume curves (**I**) for mice bearing Hepa1-6 tumors treated with PRV-LAV (1 × 10.^7^ PFUs, intratumorally). Changes in the tumor volumes are expressed as the mean ± SEM values (*n* = 9). In (**I**), statistical analysis was performed by repeated measure ANOVA. *P < 0.05; **P < 0.01; ***P < 0.001; ****P < 0.0001. Changes in the tumor volumes curves of each mouse were shown in (**J**)

Previous studies have shown that oncolytic viruses can reprogram the tumor microenvironment (TME) from an immunologically naïve (“cold”) to an inflamed (“hot”) state. Using our established Hepa1-6 model, we sought to explore the immunological mechanisms of PRV-LAV action. Tumor-infiltrating lymphocytes from control and treatment groups were isolated and subjected to CyTOF analysis. We designed a staining panel with 36 markers to comprehensively examine the tumor-infiltrating lymphocyte (TIL) populations. This panel included non-T cell lineage markers (e.g., CD11b, CD11c, CD19 and NK1.1), T cell differentiation markers (e.g., CD44, CD62L, Ly6C and FOXP3), and T cell activation and inhibition markers (e.g., PD1, TIM3, CTLA4 and LAG3). PhenoGraph analysis of the expression profiles of the 36 cell markers identified 19 main immune cell clusters: CD4^+^ T cells (cluster 9), CD8^+^ T cells (cluster 3, 4, 18), B cells (cluster 1), NK cells (cluster 7), monocytes (cluster 2, 10, 12, 17), neutrophils (cluster 8, 11, 15, 19), macrophages (cluster 5), dendritic cells (cluster 6, 16), double negative (DN) T cells (cluster 13), and non-lymphocytes (cluster 14) (Fig. 5A-B, Table S4). Through statistical analysis of the cell numbers of different cell subsets in the treatment and control groups, we found clear changes in the proportions of exhausted CD8^+^ T cells (CD8^+^ PD1^High^ LAG3^High^ Tim3^High^) (cluster 4), activated CD8^+^ T cells (CD8^+^ Tbet^+^) (cluster 3), B cells (CD19^+^ MHC II^+^) (cluster 1), naïve neutrophils (CD11b^+^ Ly6G^+^ Ly6C^+^ CD86^−^) (cluster 15), and activated neutrophils (CD11b^+^ Ly6G^+^ Ly6C^+^ CD86^+^) (cluster 11) (Fig. 5C-E). By assessing cell marker expression of different cell subsets in the treatment and control groups, we found a significant decrease in the expression of immunosuppressive molecules PD1 and TIM3, along with a significant increase in the expression of granzyme B associated with cytotoxic activity for T cells in activated CD8 T cells after PRV-LAV treatment (Fig. S4). These findings suggest that an increase in the proportion of tumor-infiltrating CD8^+^ T cells may shift the tumor environment from an immunosuppressive to an immunostimulatory state. Granzyme B expression was also increased in the exhausted CD8^+^ T cells (Fig. S5), naïve neutrophils, and activated neutrophils. The expression of MHC II was decreased in the exhausted CD8^+^ T cells and activated neutrophils (Fig. S5).

To further verify this phenomenon, tumor-infiltrating lymphocytes were isolated and analyzed by multicolor flow cytometry (Fig. S6). Compared with the mock group, PRV-LAV treatment led to a significant increase in the total number of lymphocytes in the Hepa1-6 tumors (Fig. S7A). There was no significant change in the number of dendritic cells, macrophages, monocytes, or NK cells (Fig. 5F). Furthermore, the PRV-LAV treatment led to an increase in the number of CD4^+^ T cells, CD8^+^ T cells, and neutrophils as compared with the mock group (Fig. 5F). Of these changes, the most significant was the rise in the number of CD8^+^ T cells. To analyze T cell function, we measured the mean fluorescence intensity of markers involved in T cell activation and inhibition. Dendritic cells are the most potent antigen-presenting cells (APC) to elicit naive T-cell activation. T cell activation requires important costimulatory pathway activity that involves interactions between CD28 and B7 ligands, CD80 and CD86. Indeed, we showed a significant increase in CD86 expression on dendritic cells in the PRV-LAV treatment group compared with the mock group (Fig. 5G). These findings suggest that dendritic cells in the PRV-LAV treatment group more effectively activated T cell-mediated anti-tumor function than those in the control.

Simultaneously, we found a significant decrease in the expression of immunosuppressive markers CTLA4 and TIM3 on CD4^+^ T cells, and CTLA4, PD1, TIM3, and LAG3 (*p* = 0.078) on CD8^+^ T cells (Fig. 5H); these findings are mostly consistent with CyTOF analysis, and imply that CD4^+^ and CD8^+^ T cells effectively relieve the immunosuppressive state during PRV-LAV treatment. To verify this, antibodies against mouse CD4 and CD8 were used to knock out CD4^+^ and CD8^+^ T cells, respectively. We found that knocking out of CD8^+^ T cells was able to almost abolish the therapeutic activity of PRV-LAV in the Hepa1-6 syngeneic model, with CD4^+^ T cell knockout having a less severe effect (Fig. 5I-J). These experiments confirm that PRV-LAV treatment can relieve the immune suppression of tumor-infiltrating CD8^+^ T cells and exert its anti-tumor function to clear tumor tissues.

**Fig. 6:**
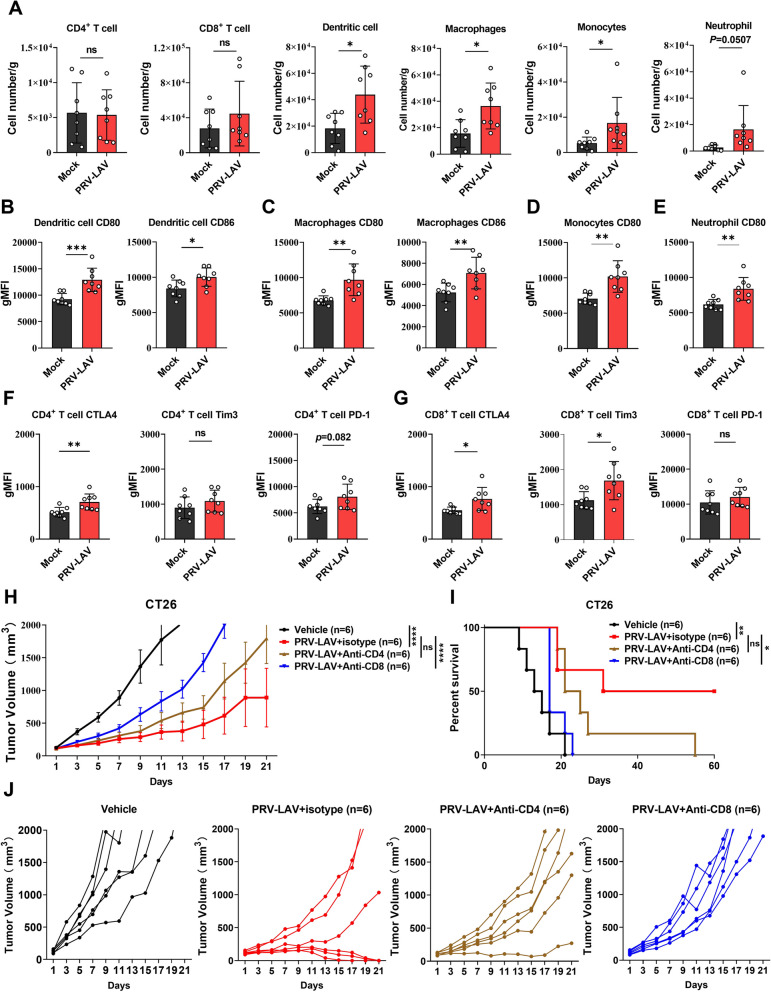
The oncolytic efficacy of PRV-LAV depends on CD8^+^ T cells in a CT26 mouse model. **A-G** Tumor immune infiltrates were analyzed by flow cytometry on the day after mice received the final of 4 doses of vehicle (*n* = 8) or PRV-LAV (*n* = 8) treatment. The numbers of tumor-infiltrating CD4^+^ T cells, CD8^+^ T cells, dendritic cells, macrophages, monocytes, and neutrophils per gram tumor were calculated (**A**). The mean fluorescence intensities of CD80 and CD86 on dendritic cells (**B**) and macrophages (**C**) were determined, as was the mean fluorescence intensity of CD80 on monocytes (**D**) and neutrophils (**E**). The mean fluorescence intensities of CTLA4, PD-1, and TIM3 on CD4^+^ T cells (**F**) and CD8^+^ T cells (**G**) were determined. Data are presented as the mean ± s.d. values. The black bars indicate the mean values. A *t* test was used to determine the significance of differences. *P < 0.05; **P < 0.01; ***P < 0.001; ****P < 0.0001. **H-J** Knockout of CD4^+^ and CD8^+^ T cells during PRV-LAV treatment. Changes in the tumor volume curves (**H**) and Kaplan–Meier survival curves (**I**) for mice bearing CT26 tumors treated with PRV-LAV (1 × 10.^7^ PFUs, intratumorally). Changes in the tumor volume curves for each mouse are shown in (**J**). Changes in the tumor volumes are expressed as the mean ± SEM values (*n* = 6). In (**H**), statistical analysis was performed by repeated measure ANOVA. Statistical analysis was performed using the log-rank test in (**I**). *P < 0.05; **P < 0.01; ***P < 0.001; ****P < 0.0001

Tumor-infiltrating lymphocytes were isolated and analyzed by multicolor flow cytometry in the CT26 syngeneic model. We found no significant difference in the total number of lymphocytes (Fig. S7B), or the numbers of CD4^+^ T cells and CD8^+^ T cells (Fig. 6A). However, compared with the mock group, we measured a significantly higher proportion of dendritic cells, macrophages, monocytes, and neutrophils in the PRV-LAV treatment group (Fig. 6A). For instance, we found a significant increase in CD80 expression on dendritic cells, macrophages, monocytes, and neutrophil (Fig. 6B-E), which may contribute to activating the functions of T cells. CD86 was also significantly higher on dendritic cells (Fig. 6B). However, immunosuppressive markers on T cells also displayed a significant rising trend. For instance, the expression of CTLA4 was significantly higher on CD4^+^ and CD8^+^ T cells (Fig. 6F-G), as was TIM3 on CD8^+^ T cells (Fig. 6F-G). PD1 also showed a trend toward increased expression; albeit this was not significant (Fig. 6F-G).

In light of these findings, we next carried out CD4^+^ and CD8^+^ T cell knockout experiments to verify the function of T cells during PRV-LAV treatment. We found that CD8^+^ T cell knockout can almost abolish the therapeutic activity of PRV-LAV in the CT26 syngeneic model, whereas the knockout of CD4^+^ T cells had less severe effects (Fig. 6H-J). These findings confirmed that the therapeutic activity of PRV-LAV in vivo was mainly dependent on CD8^+^ T cells. Although CD80 and CD86 were highly expressed in multiple tumor-infiltrating lymphocytes, the lower proportions of tumor-infiltrating lymphocytes in the CT26 tumors as compared with the Hepa1-6 tumors made APC-mediated T cell activation less convenient. Furthermore, the elevated expression of immunosuppressive markers on T cells further affected T cell activation. Thus, T cells were not fully activated, and this may explain the lower oncolytic efficacy in the CT26 syngeneic model as compared with the Hepa1-6 syngeneic model.

**Fig. 7:**
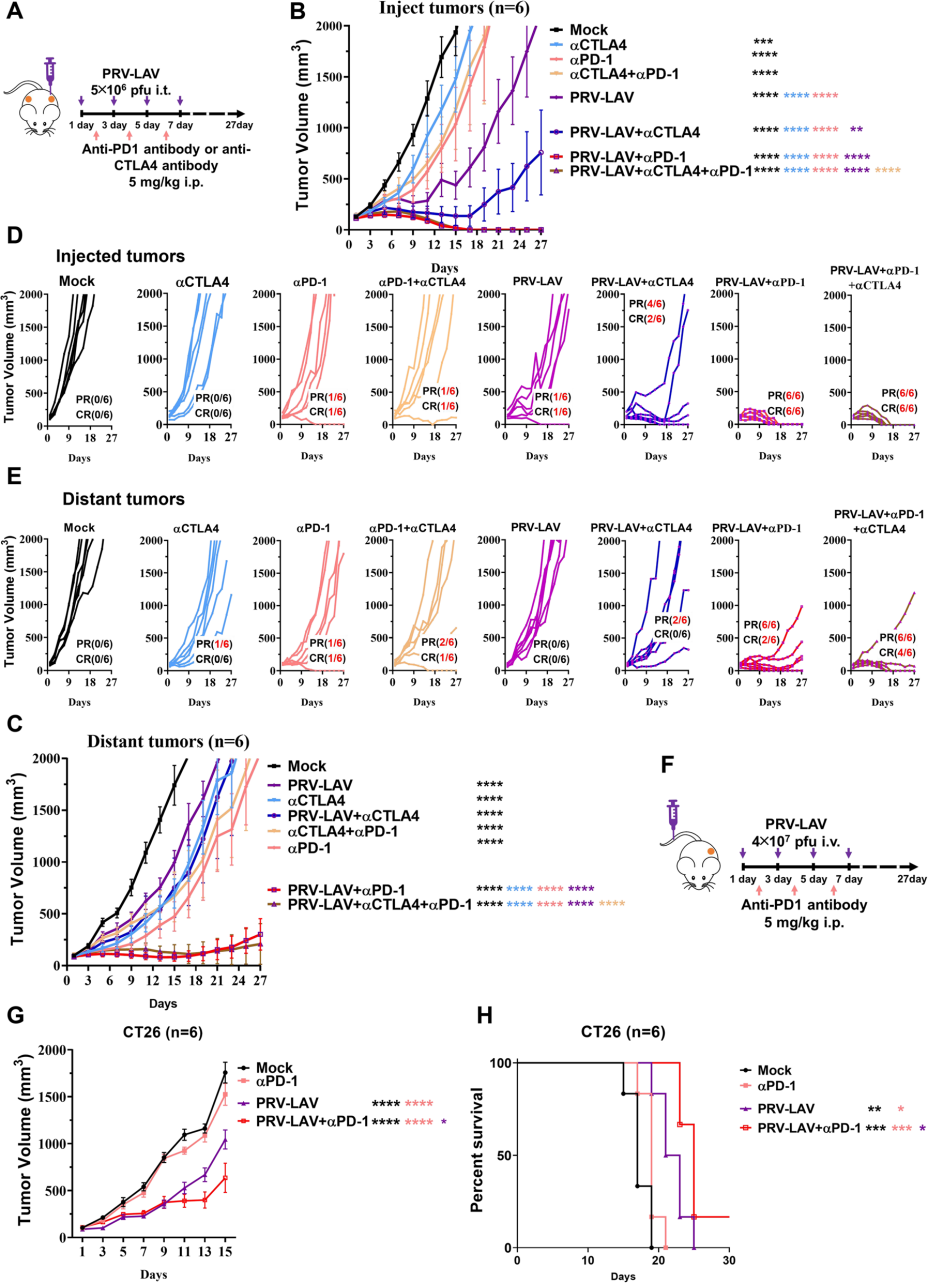
Combination of PRV-LAV with immune checkpoint inhibitors. **A** Timeline of the experimental setup. **B-E** Changes in the injected (**B**) and distant (**C**) tumor volumes curves for mice bearing CT26 tumors treated with vehicle, anti-PD1 antibody (5 mg/kg, intraperitoneally), anti-CTLA4 antibody (5 mg/kg, intraperitoneally), PRV-LAV (5 × 10^6^ PFUs, intratumorally), and or different combinations. Changes in the tumor volumes are expressed as the mean ± SEM values (*n* = 6). In (**B**) and (**C**), statistical analysis was performed by repeated measure ANOVA. *P < 0.05; **P < 0.01; ***P < 0.001; ****P < 0.0001. Changes in the injected (**D**) and distant (**E**) tumor volume curves for each mouse are shown in (**B**-**C**). Complete response (CR) means the tumor has been cleared during the observation period. Partial response (PR) means tumor growth has been inhibited and less than 70% of the maximum tumor volume during the observation period. **F** Timeline of the experimental setup. **G-H** Changes in the tumor volume curves (**G**) and Kaplan–Meier survival curves (**H**) for mice bearing CT26 tumors treated with vehicle, anti-PD1 antibody (5 mg/kg, intraperitoneally), PRV-LAV (4 × 10.^7^ PFUs, intravenously), and the combination. Changes in the tumor volumes are expressed as the mean ± SEM values (*n* = 6). In (**G**) statistical analysis was performed by repeated measure ANOVA. Statistical analysis was performed using the log-rank test in (**H**). *P < 0.05; **P < 0.01; ***P < 0.001; ****P < 0.0001

Finally, to verify the effect of the T cell activation state on the oncolytic efficacy of PRV-LAV, we used the immune checkpoint inhibitor anti-mouse PD1 or CTLA4 antibody to exacerbate T cell activity in the presence of PRV-LAV (Fig. 7A). Through this analysis, the combination of anti-mouse CTLA4 antibody with PRV-LAV was able to increase the therapeutic response against the injected tumor. We verified that relieving T cell immunosuppression can improve the therapeutic activity of PRV-LAV. However, this phenomenon has not been observed in distant tumors. Interestingly, irrespective of whether the cells were injected or the target was a distant tumor, we observed a significant synergistic effect of the combination of anti-mouse PD1 antibody and PRV-LAV. In addition, in injected tumors, there was an increase in the partial response (PR) and complete response (CR) rates from 16.7% to 100%, whereas, in the distant tumors, there was an increase in the PR rate from 16.7% (anti-mouse PD1 antibody) and 0% (PRV-LAV) to 100%, and a smaller increase in the CR rate from 16.7% (anti-mouse PD1 antibody) and 0% (PRV-LAV) to 33.3%. Administering the combination of anti-PD1 antibody, anti-CTLA4 antibody, and PRV-LAV further improved the therapeutic activity against the distant tumors, increasing the CR rate to 66.7% (Fig. 7B-E). Furthermore, the combination of PRV-LAV by intravenous injection with anti-mouse PD1 antibody was evaluated. PRV-LAV intravenous treatment alone significantly inhibited tumor growth, and combination with anti-mouse PD1 antibody also exhibited a synergistic effect, extending survival of the mice with tumors (Fig. 7F-H).

## Discussion

In screens of novel oncolytic virus strains, reagents with strong oncolytic activity were the first to be considered, and oncologists subsequently devoted much time and effort to obtaining a sufficiently safe oncolytic virus strain [27–29]. This process, however, was fraught with challenges and uncertainties. Yet, if we assume that oncolytic virus candidates possess good safety, we need only prove the tumoricidal ability of candidates in vitro and in vivo. This substantially improves the efficiency of screening for novel oncolytic viruses. But how can virus strain safety be evaluated? Vaccinologists have a satisfactory answer: live-attenuated vaccines are not only on the market but have been widely confirmed by massive amounts of data [30, 31]. Thus, so long as the tumoricidal abilities of these vaccines are confirmed, candidates can quickly be converted to novel antineoplastic drugs for cancer treatment [32]. Here, we demonstrated that PRV-LAV selectively kills tumor cells by inducing cancer cell death, and was well tolerated in mice and rats. Thus, it may be an effective oncolytic agent to treat malignant tumors.

Oncotherapeutic research on PRV is infrequently reported [33, 34]. A conditionally replicating PRV for HER2/neu-overexpressing bladder cancer therapy showed that PRV offered tumor-suppressive activity against bladder cancer in vitro and in vivo via an unclear mechanism [35]. However, although this PRV virus carried both the gD and HSV-1 TK genes under transcriptional control of the HER-2/neu promoter, it had lower antitumor activity in vitro than the PRV-LAV HB2000 strain evaluated in this paper. Both PRV and HSV belong to the *Alphaherpesvirinae* subfamily. Yet, whether the oncolytic activity of PRV is similar to that of HSV is unknown. In this study, we compared the oncolytic activity of PRV and HSV against 38 cancer cell lines. The tumoricidal activity of HB2000 was not similar to that of HSV but was complementary, a finding that may provide new insight into patients who have a low response to HSV agent treatment. Cocktail therapy—the combination of PRV with other HSVs or oncolytic viruses—may expand the range of patients able to be treated with oncolytic agents and significantly prolong patient survival.

The molecular mechanism of tumor tropism is important for predicting the antitumor efficacy of oncolytic viruses. Although an increasing number of oncolytic agents have been developed, few reports have elucidated the mechanism(s) of tumor tropism: oncolytic poliovirus recognizes the tumor-overexpressed CD155 [36–38]; reovirus preferentially replicates in cells with activated oncogenic Ras signaling [39–41]; vesicular stomatitis virus (VSV) requires defects in the IFN pathway [42–44]; and M1 requires ZAP deficiency [7, 45]. Herein, we showed that the EGFR signaling pathway regulates PRV infection and proliferation. EGFR was previously reported to be the receptor for human cytomegalovirus (HCMV) [46, 47] but not for HSV [48]. Others confirmed that NRP1 can contribute to the binding with CendR, which is found in many viruses, including EBV [49], and SARS-CoV2 [50, 51]. However, PRV gB possesses the same furin splice site (RRAR) as SARS-CoV2, which suggests potential interaction of gB with NRP1. Indeed, we found that the interaction between PRV and EGFR depends on NRP1, as it mediates virus entry into cancer cells via macropinocytosis and lipid raft-dependent endocytosis. NRP1 and EGFR were highly expressed in most cancer cells [52–58], and this suggests that PRV-LAV may have potential utility in personalized cancer therapy among patients with NRP1/EGFR-overexpressing tumors. Although EGFR inhibitors have been widely used for the treatment of malignant tumors, they do not influence the activity of PRV oncolytic agents as a novel therapy against EGFR-overexpressing tumors. Because PRV oncolytic agents can stimulate specific antitumor immunity, they effectively inhibit tumor recurrence and metastasis, which may not be restored by small-molecule EGFR inhibitors. Cocktail therapy may thus provide new insight into cancer treatment.

In early research, PRV was demonstrably safe in higher primates and humans. However, after the first case of human infectious endophthalmitis caused by PRV infection in 2017, the possibility of cross-species transmission from swine to human was confirmed [59]. To date, about 20 patients have been reported as having PRV infection in China as a result of a suspected cross-species transmission [60–63]. Additional investigations identified the genotype of the virus as clade 2.2, which may be associated with the PRV (clade2.2) outbreak in swine within the Shangdong province of China in 2012 [17]. The genotype of PRV-LAV HB2000 is clade 2.1. As yet, there has not been any human diseases caused by this clade. Furthermore, extensive studies reveal the deletions of gE, gI, and TK can significantly improve the safety of PRV in vivo [23, 64, 65]. This knowledge will provide a basis for the safety of PRV-LAV HB2000 in cancer therapy in humans.

Previous studies showed that oncolytic viruses can reprogram the tumor microenvironment and increase the infiltration of immune cells; indeed, T-VEC can reprogram immune-silent tumors into immune-inflamed tumors and induce the expression of PD1 and PDL1. Combining MEK inhibition and anti-PD1 antibody treatment with T-VEC can exacerbate this response [66]. Oncolytic HSV OVH can reduce the populations of Treg cells and MDSCs, and increase the population of antitumor immune cells. The combination of an anti-PD1 antibody with another immune checkpoint inhibitor, anti-TIGIT antibody, can also improve the therapeutic potency of OVH [25]; as does treatment with NDV and Maraba rhabdovirus [25, 67]. Given that PRV can induce cancer cell death via apoptosis through ER stress, this begs the question: Can PRV treatment induce different tumor microenvironments? We demonstrated that PRV treatment can enhance the infiltration of CD4^+^ T cells, CD8^+^ T cells and neutrophils into a Hepa 1–6 tumor microenvironment. Furthermore, we noted a decrease in the expression of immunosuppressive markers, such as PD1, CTLA4, and TIM3, on T cells. However, this phenotype did not occur in the CT26 model: reduced lymphocytic infiltration was seen to lower the proportion of APC cells and T cells present per unit volume, and this, in turn, likely reduced the interactions between APC cells and T cells for T cell activation.

Immune checkpoint inhibitors can increase the activation of T cells, and, when delivered in combination with anti-PD1 antibody, can significantly enhance the therapeutic activity of PRV-LAV. The combination of multiple immune checkpoint inhibitors would then likely lead to a further increase in T cell activity. Thus, a combination of inhibitors with PRV may significantly improve the therapeutic activity of PRV, and be tailored for the benefit of more cancer patients.

We also observed the significant rise of naïve and activated neutrophils in the Hepa1-6 model post PRV-LAV treatment. Neutrophils can secrete the elastase to kill genetically diverse cancer cells [68]. Thus, neutrophils may improve the therapeutic activity of PRV-LAV through this process.

Based on the aforementioned findings, it is evident that PRV-LAV therapy can rapidly eliminate gliomas and liver cancer tumors, possibly due to the involvement of NRP1 and EGFR. It is worth noting that these two molecules are frequently overexpressed in several cancer types, including lung, colon, head and neck, brain, liver, and pancreatic cancers [69, 70]. Therefore, PRV-LAV may exhibit stronger targeting specificity and therapeutic efficacy in these specific cancer types. Consequently, when PRV-LAV is administered intravenously, it can accumulate in these tumor tissues and serve as a gene vector to deliver immune checkpoint inhibitors, cytokines, tumor suppressor genes, and other therapeutic agents, thereby enhancing the anti-tumor effects. Furthermore, the tumor-specific replication of PRV-LAV promotes lymphocyte infiltration into the tumor microenvironment and alleviates the immune inhibitory status of T cells. Therefore, the combination of PRV-LAV and chimeric antigen receptor T-cell (CAR-T) immunotherapy [71] is expected to significantly enhance the infiltration of CAR-T cells into solid tumors and counteract tumor-induced immune suppression, thereby significantly boosting the therapeutic responses of CAR-T cells against these solid tumors. Therefore, these therapeutic approaches have the potential to benefit patients suffering from these malignancies.

## Conclusion

In summary, we established an effective strategy to screen oncolytic viruses from live-attenuated vaccines and confirmed that the tumor tropism of PRV-LAV was associated with the NRP1/EGFR signaling pathway. We also confirmed that PRV-LAV can selectively induce cancer cell death via apoptosis of ER stress signaling pathways. PRV-LAV treatment was found to completely clear tumor tissues in nude mice and immunocompetent mice. PRV-LAV also reprogrammed immune-silent tumors into immune-inflamed tumors, and showed that the therapeutic potency of PRV-LAV could be improved when combined with the delivery of an appropriate regimen of an anti-PD1 antibody. Collectively, our research highlights an efficient personalized therapy via NRP1/EGFR signaling of malignant tumors. It is foreseeable that oncolytic PRV-LAV will become an important tool in the oncotherapeutic arsenal for humans.

### Supplementary Information

**Additional file 1: Table S1.** The list of cell lines. **Table S2.** The list of antibodies for CyTOF analysis. **Table S3.** Kinase inhibitors with inhibition rates above 80% in GBM and PK-15 cells. **Fig. S1.** PRV proliferation is associated with EGFR signaling. **Fig. S2.** PRV-LAV gB interacts with EGFR via NRP1. **Fig. S3.** PRV-LAV treatment was well tolerated in mice and rats. **Table S4.** The phenotypes of 19 kinds of clusters. **Fig. S4.** Mean fluorescence intensity of markers in cluster 3 (activated CD8+ T cell). **Fig. S5.** Mean fluorescence intensity of markers in cluster 4 (exhausted CD8+ T cell). **Fig. S6.** The gating strategy of immunological mechanism. **Fig. S7.** The total number of lymphocytes in Hepa1-6 and CT26 tumor post mock or PRV-LAV treatment.

## Data Availability

The datasets used and/or analyzed during the current study are available from the corresponding author upon reasonable request.
