# Correction: The potential of swine pseudorabies virus attenuated vaccine for oncolytic therapy against malignant tumors

**DOI:** 10.1186/s13046-023-02891-y

**Published:** 2023-11-22

**Authors:** Guosong Wang, Jiali Cao, Mengxuan Gui, Pengfei Huang, Liang Zhang, Ruoyao Qi, Ruiqi Chen, Lina Lin, Qiangyuan Han, Yanhua Lin, Tian Chen, Peiqing He, Jian Ma, Rao Fu, Junping Hong, Qian Wu, Hai Yu, Junyu Chen, Chenghao Huang, Tianying Zhang, Quan Yuan, Jun Zhang, Yixin Chen, Ningshao Xia

**Affiliations:** 1 State Key Laboratory of Vaccines for Infectious Diseases, National Institute of Diagnostics and Vaccine Development in Infectious Diseases, State Key Laboratory of Molecular Vaccinology and Molecular Diagnostics, Collaborative Innovation Center of Biologic Products National Innovation Platform for Industry-Education Intergration in Vaccine ResearchSchool of Life Sciences, School of Public Health, Xiang An Biomedicine Laboratory, Xiamen University, Xiamen, People’s Republic of China; 2 Department of Laboratory Medicine, Fujian Key Clinical Specialty of Laboratory Medicine, Women and Children’s Hospital, School of Medicine, Xiamen University, Xiamen, People’s Republic of China

**Correction:** ***J Exp Clin Cancer Res*** **42, 284 (2023)**

**https://doi.org/10.1186/s13046-023-02848-1**

Following publication of the original article [1], errors were identified by author in the body and Figs. 2 and 4, specifically:

1. In Fig. 2c, there is a lack of antigen for Wb detection, despite the description of relevant experiments in the legend. However, after correction, the readers will have a more intuitive understanding.
2. In Fig. 2a, the color marking of the top green dot is incorrect, it should be blue.
3. In Fig. 4g, the mice control group was mistakenly recorded twice resulting to a total of 6, it should be 5.
4. In the main text, the tumor clearance efficiency of PRV-LAV in the CT26 model is 50%, not 40%.
5. In the legend of Fig. 5, the number of mouse in Fig. 5I is (*n* = 9), not (*n* = 6).
6. In the description of animal experiments in the Materials and Methods section, the sentence “Age matched (14 to 16-week-old) naïve mice were used as controls (*n* = 5)” should be (*n* = 6).

**Fig. 2:**
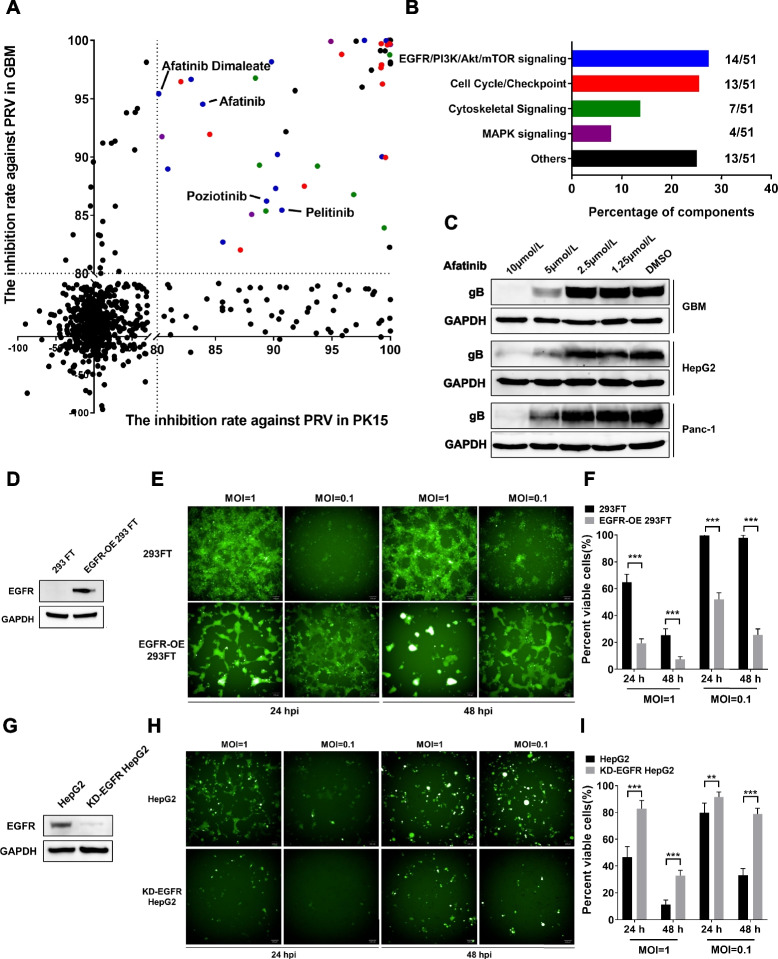
The expression of EGFR regulates the proliferation of PRV-LAV. **A**, **B** GBM cells and PK-15 cells were pretreated with vehicle or 5 μM kinase inhibitor and were then infected with PRV-LAV-mNeonGreen (MOI = 0.001). The inhibition rates against PRV infection in GBM and PK-15 cells were calculated by Harmony imaging and analysis software. The results are presented on a scatter plot. Each point on the plot represents a kinase inhibitor (**A**). Kinase inhibitors with inhibition rates of at least 80% in both GBM and PK-15 cells were classifed according to their molecular function in signaling pathways (**B**). This experiment was repeated three times. **C** The expression of PRV gB was analyzed by western blotting after cancer cells were pretreated with the EGFR inhibitor afatinib and then infected with PRV-LAV HB2000 (GBM, MOI = 0.001; HepG2 and Panc-1, MOI = 0.01) and cultured for 48 h. **D** The expression of EGFR in 293FT and EGFR-OE 293FT cells was analyzed by western blotting. **E**, **F** 293FT and EGFR-OE 293FT cells were infected with PRV-LAV-mNeonGreen (MOI = 0.1, 1). Phase-contrast and fuorescence micrographs were acquired with an Opera Phenix High Content Screening System (**E**), with cell viability assays were performed (**F**) 24 h and 48 h post-infection. E Scale bars, 100 μm. **F** Data are presented as the mean ± s.d. values (*n* = 6). 293FT cells vs. EGFR-OE 293FT cells. A t test was used to determine the signifcance of diferences in the percentages of viable cells post-viral infection. **G-I** Knockdown of EGFR expression in HepG2 cells suppressed the proliferation of PRV-LAV. The expression of EGFR in HepG2 and KD-EGFR HepG2 cells was analyzed by western blotting (**G**). HepG2 and KD-EGFR HepG2 cells were infected with PRV-LAV-mNeonGreen (MOI = 0.1, 1). Phase-contrast and fuorescence micrographs were acquired with an Opera Phenix High Content Screening System (**H**), with cell viability assays were performed (**I**) 24 h and 48 h post-infection. Scale bars, 100 μm. The data are presented as the mean ± s.d. values. Data are presented as the mean ± s.d. values (*n* = 6). The black bars indicate the mean values. A *t* test was used to determine the signifcance of diferences in the percentages of viable cells post-viral infection

**Fig. 4:**
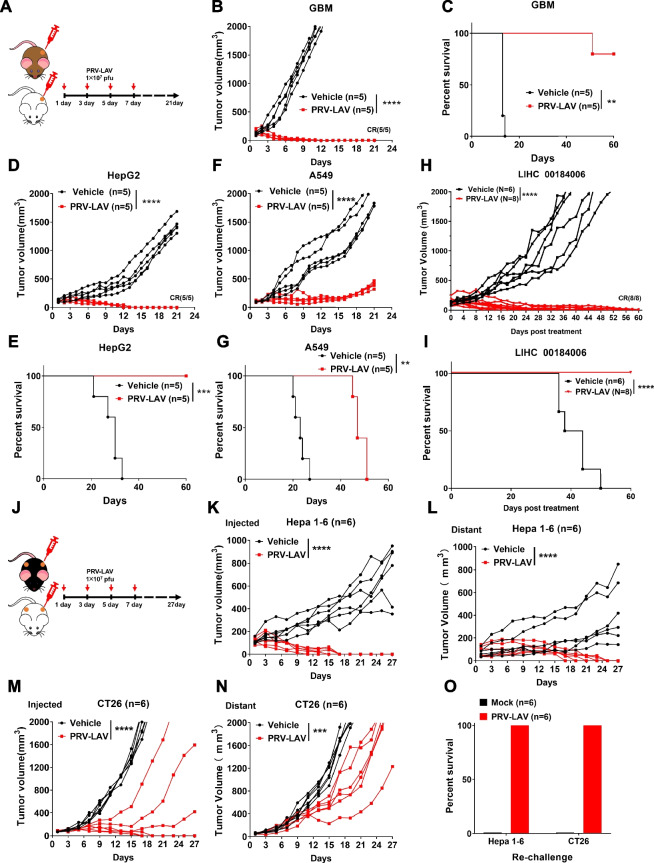
In vivo therapeutic efficacy of PRV-LAV. **A** Timeline of the experimental setup for the experiments in the Balb/c nude or NOD-scid mouse model. **B-G** Tumor volume curves (**B**, **D**, **F**) and Kaplan–Meier survival curves (**C**, **E**, **G**) for mice bearing GBM, HepG2, and A549 tumors treated with vehicle or PRV-LAV (1 × 107 PFUs, intratumorally). **H-I** Therapeutic activity of PRV-LAV in the liver cancer PDX model (LIHC 00184006). Tumor volume curves (**H**) and Kaplan–Meier survival curves (**I**) for PDX mice treated with 4 doses of vehicle (*n* = 6) or PRV-LAV (*n* = 8) (1 × 10^7^ PFUs, intratumorally). **J** Timeline of the experimental setup for the experiments in Hepa1-6 and CT26 syngeneic models in immunocompetent mice. **K-N** Changes in the injected (**K**, **M**) and distant (**L**, **N**) tumor volumes curves for mice bearing Hepa1-6 and CT26 tumors treated with vehicle or PRV-LAV (1 × 10^7^ PFUs, intratumorally). In (**B**, **D**, **F**, **H**, **K-N**), comparisons were performed by AUC analysis. Statistical analysis was performed by *t* test. *P < 0.05; **P < 0.01; ***P < 0.001; ****P < 0.0001. Statistical analysis was performed using the log-rank test in (**C**, **E**, **G**, **I**). **O** Tumor cells were inoculated subcutaneously into the single hind-flank of mice. After 60 days post PRV-LAV treatment, cured mice treated with PRV-LAV were rechallenged with two-fold increased number of the same cancer cells. Recurrence rates were monitored in all groups

The correct Figs. 2 and 4 are given below. These corrections do not affect the overall result or conclusion of the article. The original article has been corrected.
