# Supplementary material for: The potential of swine pseudorabies virus attenuated vaccine for oncolytic therapy against malignant tumors
Source: J Exp Clin Cancer Res. 2023 Oct 27;42:284. doi: 10.1186/s13046-023-02848-1 (PMC10604416; doi:10.1186/s13046-023-02848-1)
Supplement: Supplementary file 1 — Additional file 1: Table S1. The list of cell lines. Table S2. The list of antibodies for CyTOF analysis. Table S3. Kinase inhibitors with inhibition rates above 80% in GBM and PK-15 cells. Fig. S1. PRV proliferation is associated with EGFR signaling. Fig. S2. PRV-LAV gB interacts with EGFR via NRP1. Fig. S3. PRV-LAV treatment was well tolerated in mice and rats. Table S4. The phenotypes of 19 kinds of clusters. Fig. S4. Mean fluorescence intensity of markers in cluster 3 (activated CD8+ T cell). Fig. S5. Mean fluorescence intensity of markers in cluster 4 (exhausted CD8+ T cell). Fig. S6. The gating strategy of immunological mechanism. Fig. S7. The total number of lymphocytes in Hepa1-6 and CT26 tumor post mock or PRV-LAV treatment. [file 13046_2023_2848_MOESM1_ESM.docx]

Supplementary Materials for

**The potential of swine pseudorabies virus attenuated vaccine for oncolytic therapy against malignant tumors**

Guosong Wang^a1^, Jiali Cao^b1^, Mengxuan Gui^a1^, Pengfei Huang^a1^, Liang Zhang^a1^, Ruoyao Qi^a^, Ruiqi Chen^a^, Lina Lin^a^, Qiangyuan Han^a^, Yanhua Lin^a^, Tian Chen^a^, Peiqing He^a^, Jian Ma^a^, Rao Fu^a^, Junping Hong^a^, Qian Wu^a^, Hai Yu^a^, Junyu Chen^a^, Chenghao Huang^a^*, Tianying Zhang^a^*, Quan Yuan^a^*, Jun Zhang^a^*, Yixin Chen^a^*, Ningshao Xia^a^*

^a^ State Key Laboratory of Vaccines for Infectious Diseases, National Institute of Diagnostics and Vaccine Development in Infectious Diseases, State Key Laboratory of Molecular Vaccinology and Molecular Diagnostics, Collaborative Innovation Center of Biologic Products，National Innovation Platform for Industry-Education Intergration in Vaccine Research，School of Life Sciences, School of Public Health, Xiang An Biomedicine Laboratory，Xiamen University, Xiamen, People's Republic of China

^b^ Department of Laboratory Medicine, Fujian Key Clinical Specialty of Laboratory Medicine, Women and Children’s Hospital, School of Medicine, Xiamen University,Xiamen, People's Republic of China

*Corresponding author. E-mail:[yxchen2008@xmu.edu.cn](mailto:yxchen2008@xmu.edu.cn) (Y.C.), [nsxia@xmu.edu.cn](mailto:nsxia@xmu.edu.cn) (N.X.), [zhangj@xmu.edu.cn](mailto:zhangj@xmu.edu.cn) (J.Z.), [yuanquan@xmu.edu.cn](mailto:yuanquan@xmu.edu.cn) (Q.Y.), [huangchenghao@xmu.edu.cn](mailto:huangchenghao@xmu.edu.cn) (C.H.), [tyzhang1003@163.com](mailto:tyzhang1003@163.com) (T.Z.)

^1^ These authors contributed equally to this work.

**This PDF file includes:**

Table S1. The list of cell lines.

Table S2. The list of antibodies for CyTOF analysis.

Table S3. Kinase inhibitors with inhibition rates above 80% in GBM and PK-15 cells.

Fig. S1. PRV proliferation is associated with EGFR signaling.

Fig. S2. PRV-LAV gB interacts with EGFR via NRP1.

Fig. S3. PRV-LAV treatment was well tolerated in mice and rats.

Table S4. The phenotypes of 19 kinds of clusters.

Fig. S4. Mean fluorescence intensity of markers in cluster 3 (activated CD8+ T cell).

Fig. S5. Mean fluorescence intensity of markers in cluster 4 (exhausted CD8+ T cell).

Fig. S6. The gating strategy of immunological mechanism.

Fig. S7. The total number of lymphocytes in Hepa1-6 and CT26 tumor post mock or PRV-LAV treatment.

**Table S1. The list of cell lines.**

| **Cell** | **Sources** | **Identifier** |
| --- | --- | --- |
| **A549** | ATCC | CCL-185 |
| **NCI-H1299** | ATCC | CRL-5803 |
| **NCI-H1975** | ATCC | CRL-5908 |
| **H661** | ATCC | HTB-183 |
| **EBC-1** | Thermo Fisher Scientific | 11875101 |
| **DMS114** | ATCC | CRL-2066 |
| **BEL7404** | China National Infrastructure of Cell Line Resource | 3131C0001000700064 |
| **SMMCC7721** | China National Infrastructure of Cell Line Resource | 3111C0001CCC000087 |
| **Hep3b** | ATCC | HB-8064 |
| **huh7** | China National Infrastructure of Cell Line Resource | 3131C0001000700182 |
| **HepG2** | ATCC | HB-8065 |
| **HEPA 1-6** | ATCC | CRL-1830 |
| **ASPC-1** | ATCC | CRL-1682 |
| **CAPAN-2** | ATCC | HTB-80 |
| **PANC 10.5** | ATCC | CRL-2547 |
| **PANC-1** | ATCC | CRL-1469 |
| **A498** | ATCC | HTB-44 |
| **RENCA** | ATCC | CRL-2947 |
| **CNE1** | a gift from Prof. Honglin Chen |  |
| **CNE2** | a gift from Prof. Honglin Chen |  |
| **SIHA** | ATCC | HTB-35 |
| **CASKI** | ATCC | CRL-1550 |
| **TOV-112D** | Xiamen Jika Technology Co., Ltd | TOV-112D |
| **SKOV-3** | ATCC | HTB-77 |
| **HCT116** | ATCC | CCL-247 |
| **SW1116** | Shanghai YSRIBIO industrial co., LTD. |  |
| **AGS** | ATCC | CRL-1739 |
| **SGC7901** | ATCC | GDC150 |
| **BGC823** | ATCC | GDC151 |
| **A20** | ATCC | TIB-208 |
| **RAJI** | ATCC | CCL-86 |
| **Hep2** | ATCC | CCL-23 |
| **GBM** | isolated from glioma tissue |  |
| **B16F10** | ATCC | CRL-6475 |
| **MCF7** | China National Infrastructure of Cell Line Resource |  |
| **U2OS** | ATCC | HTB-96 |
| **HFF-1** | ATCC | SCRC-1041 |
| **HPNE** | ATCC | CRL-4023 |
| **HepaRG** | Life Technologies | HPRGC10 |
| **iehESCs** | a gift from Xiamen Immocell Biotechnology Co.,Ltd. |  |
| **HSF** | a gift from Xiamen Immocell Biotechnology Co.,Ltd. |  |
| **PHH** | Liver Biotechnology (Shenzhen) Co., Ltd | LV-PHH001 |

**Table S2. The list of antibodies for CyTOF analysis.**

| REAGENT or RESOURCE | SOURCE | IDENTIFIER |
| --- | --- | --- |
| Antibodies | | |
| 161Dy-Tbet (Clone 4B10) | Fluidigm | Cat# 3161014B |
| X152Sm-CD3e (Clone 145-2C11) | Fluidigm | Cat# 3152004B |
| X165Ho-CD31 (Clone 390) | Fluidigm | Cat# 3165013B |
| X143Nd-TCRb (Clone H57-597) | Fluidigm | Cat# 3143010B |
| X167Er-GATA3 (Clone TWAJ) | Fluidigm | Cat# 3167007A |
| X145Nd-CD4 (Clone RM4-5) | Fluidigm | Cat# 3145002B |
| X169Tm-CD206 (Clone C068C2) | Fluidigm | Cat# 3169021B |
| X164Dy-TGFb (Clone TW7-16B4) | Fluidigm | Cat# 3164014B |
| X163Dy-Bcl6 (Clone K112-91) | Fluidigm | Cat# 3163012B |
| X158Gd-FoxP3 (Clone FJK-16s) | Fluidigm | Cat# 3158003A |
| X170Er-NK1.1 (Clone PK136) | Fluidigm | Cat# 3170002B |
| X160Gd-CD62L (Clone MEL-14) | Fluidigm | Cat# 3160008B |
| X156Gd-CD14 (Clone Sa14-2) | Fluidigm | Cat# 3156009B |
| X141Pr-Ly6G (Clone 1A8) | Fluidigm | Cat# 3141008B |
| X209Bi-MHC-II (Clone M5/114.15.2) | Fluidigm | Cat# 3209006B |
| X162Dy-TIM3 (Clone RMT3-23) | Fluidigm | Cat# 3162029B |
| X159Tb-PD1 (Clone J43) | Fluidigm | Cat# 3159023B |
| X174Yb-LAG3 (Clone C9B7W) | Fluidigm | Cat# 3174019B |
| X172Yb-CD86 (Clone GL1) | Fluidigm | Cat# 3172016B |
| X153Eu-PDL1-EQ4 (Clone 10F.9G2) | Fluidigm | Cat# 3153016B |
| X148Nd-CD11b (Clone M1/70) | Fluidigm | Cat# 3148003B |
| X194Pt-KLRG1 (Clone MAFA) | In this paper | In this paper |
| X171Yb-TIGIT (Clone Vstm3) | In this paper | In this paper |
| X176Yb-GrzB (Clone EPR20129-217) | In this paper | In this paper |
| X144Nd-CD127 (Clone A7R34) | In this paper | In this paper |
| X166Er-CD19 (Clone 1D3) | In this paper | In this paper |
| X168Er-CD8a (Clone 53-6.7) | In this paper | In this paper |
| X155Gd-CD44 (Clone IM7) | In this paper | In this paper |
| X173Yb-Ly6C (Clone HK1.4) | In this paper | In this paper |
| Purified KLRG1 (Clone MAFA) | Biolegend | Cat# 164902;  RRID: AB_2910348 |
| Purified TIGIT (Clone Vstm3) | Biolegend | Cat# 156102;  RRID: AB_2750250 |
| Purified Granzyme B (Clone EPR20129-217) | Abcam | Cat# ab245038 |
| Purified CD127 (Clone A7R34) | Biolegend | Cat# 135002;  RRID: AB_1937287 |
| Purified CD19 (Clone 1D3) | Biolegend | Cat# 152402;  RRID: AB_2629714 |
| Purified CD8a (Clone 53-6.7) | Biolegend | Cat# 100755;  RRID: AB_2562796 |
| Purified CD44 (Clone IM7) | Biolegend | Cat# 103001;  RRID: AB_312952 |
| Purified Ly6C (Clone HK1.4) | Biolegend | Cat# 128039;  RRID: AB_2563783 |

**Table S3. Kinase inhibitors with inhibition rates above 80% in GBM and PK-15 cells.**

| **Name** | **Pathways** | **Target** |
| --- | --- | --- |
| SGI-7079 | Angiogenesis; Apoptosis; Tyrosine Kinase/Adaptors | c-Met/HGFR inhibitor; c-RET inhibitor; FLT inhibitor; Src inhibitor; TAM Receptor inhibitor |
| OTS514 | MAPK | TOPK inhibitor |
| PF-00562271 | Angiogenesis; Cell Cycle/Checkpoint; Tyrosine Kinase/Adaptors; Cytoskeletal Signaling | CDK inhibitor; FAK inhibitor; PYK2 inhibitor |
| PHA767491 HCl | PI3K/Akt/mTOR signaling; Cell Cycle/Checkpoint; GPCR/G Protein; Stem Cells | CDK inhibitor; cholecystokinin inhibitor; GSK-3 inhibitor |
| Sanguinarine chloride | Angiogenesis; JAK/STAT signaling; Tyrosine Kinase/Adaptors | EGFR antagonist; VEGFR inhibitor |
| Mitoxantrone hydrochloride | DNA Damage/DNA Repair | Topoisomerase inhibitor |
| Dasatinib | Angiogenesis; Tyrosine Kinase/Adaptors; Cytoskeletal Signaling | Bcr-Abl inhibitor; c-Kit inhibitor; Ephrin Receptor inhibitor; Src inhibitor |
| PIK75 | DNA Damage/DNA Repair; PI3K/Akt/mTOR signaling | DNA-PK inhibitor; PI3K inhibitor |
| Nocodazole | Angiogenesis; Cytoskeletal Signaling | Bcr-Abl inhibitor; Microtubule Associated inhibitor |
| AP24534 Ponatinib | Angiogenesis; Tyrosine Kinase/Adaptors; Cytoskeletal Signaling | Bcr-Abl inhibitor; c-Kit inhibitor; FGFR inhibitor; PDGFR inhibitor; Src inhibitor; VEGFR inhibitor |
| RGB-286638 free base | Cell Cycle/Checkpoint | CDK inhibitor |
| Afatinib (BIBW2992) | Angiogenesis; JAK/STAT signaling; Tyrosine Kinase/Adaptors | EGFR inhibitor; HER inhibitor |
| HS-173 | PI3K/Akt/mTOR signaling | PI3K inhibitor |
| PIK-75 | DNA Damage/DNA Repair; PI3K/Akt/mTOR signaling | DNA-PK inhibitor; PI3K inhibitor |
| AP26113 | Angiogenesis; Tyrosine Kinase/Adaptors; JAK/STAT signaling | ALK inhibitor; EGFR inhibitor; IGF-1R inhibitor |
| Neratinib(HKI-272) | Angiogenesis; PI3K/Akt/mTOR signaling; JAK/STAT signaling; Tyrosine Kinase/Adaptors | EGFR inhibitor; HER inhibitor; PDK inhibitor; Src inhibitor; VEGFR inhibitor |
| Pelitinib (EKB-569) | Angiogenesis; MAPK; JAK/STAT signaling; Tyrosine Kinase/Adaptors | EGFR inhibitor; MEK inhibitor; Raf inhibitor; Src inhibitor |
| TSU-68 (SU6668, Orantinib) | Angiogenesis; Tyrosine Kinase/Adaptors | FGFR inhibitor; PDGFR inhibitor; VEGFR inhibitor |
| Afatinib (BIBW2992) Dimaleate | Angiogenesis; JAK/STAT signaling; Tyrosine Kinase/Adaptors | EGFR inhibitor; HER inhibitor |
| Dinaciclib (SCH727965) | Cell Cycle/Checkpoint | CDK inhibitor |
| LDK378(Ceritinib) | Angiogenesis; Proteases/Proteasome; Tyrosine Kinase/Adaptors | ALK inhibitor; FLT inhibitor; IGF-1R inhibitor; Serine Protease inhibitor |
| Perifosine (KRX-0401) | Cytoskeletal Signaling; PI3K/Akt/mTOR signaling | Akt inhibitor |
| WHI-P258 | Angiogenesis; Chromatin/Epigenetic; JAK/STAT signaling; Tyrosine Kinase/Adaptors; Stem Cells | EGFR inhibitor; JAK inhibitor |
| TP0903 | Tyrosine Kinase/Adaptors | TAM Receptor inhibitor |
| HMN214 | Cell Cycle/Checkpoint | PLK inhibitor |
| AZD5438 | Cell Cycle/Checkpoint | CDK inhibitor |
| PP121 | Angiogenesis; PI3K/Akt/mTOR signaling; Tyrosine Kinase/Adaptors | Hck inhibitor; mTOR inhibitor; PDGFR inhibitor; Src inhibitor; VEGFR inhibitor |
| WS3 | Angiogenesis; NF-Κb; JAK/STAT signaling; Tyrosine Kinase/Adaptors | EGFR inducer; IκB/IKK inhibitor |
| Poziotinib (HM781-36B) | Angiogenesis; JAK/STAT signaling; Tyrosine Kinase/Adaptors | HER inhibitor |
| OSI 930 | Angiogenesis; MAPK; Tyrosine Kinase/Adaptors | CSF-1R inhibitor; FLT inhibitor; Raf inhibitor; Src inhibitor; VEGFR inhibitor |
| Flavopiridol (Alvocidib) hydrochloride | Cell Cycle/Checkpoint | CDK inhibitor |
| CTx0294885 | Others | Others inhibitor |
| Pseudolaric Acid B | MAPK | p38 MAPK inhibitor |
| AT 7519 hydrochloride salt | PI3K/Akt/mTOR signaling; Cell Cycle/Checkpoint; Stem Cells | CDK inhibitor; GSK-3 inhibitor |
| Torin 2 | DNA Damage/DNA Repair; PI3K/Akt/mTOR signaling | ATM/ATR inhibitor; DNA-PK inhibitor; mTOR inhibitor |
| CH5183284 (Debio-1347) | Angiogenesis; Tyrosine Kinase/Adaptors | FGFR inhibitor |
| GNF-7 | Angiogenesis; Neuroscience; Cytoskeletal Signaling | AChR inhibitor; Bcr-Abl inhibitor |
| Rigosertib (ON-01910) | Cell Cycle/Checkpoint | PLK inhibitor |
| Geldanamycin | Cytoskeletal Signaling; Proteases/Proteasome; Metabolism | HSP inhibitor; Tyrosine Kinases inhibitor |
| Picropodophyllin (PPP) | Tyrosine Kinase/Adaptors | IGF-1R inhibitor |
| THZ1 | Cell Cycle/Checkpoint | CDK inhibitor |
| Bardoxolone Methyl | NF-Κb | IκB/IKK inhibitor |
| Staurosporine | Angiogenesis; Cytoskeletal Signaling | PKC inhibitor; Src inhibitor |
| AZD7762 | Cell Cycle/Checkpoint | Chk inhibitor |
| AT7519 | PI3K/Akt/mTOR signaling; Cell Cycle/Checkpoint; Stem Cells | CDK inhibitor; GSK-3 inhibitor |
| Anisomycin | MAPK | JNK inhibitor |
| SNS-032 (BMS-387032) | PI3K/Akt/mTOR signaling; Cell Cycle/Checkpoint; Stem Cells | CDK inhibitor; GSK-3 inhibitor |
| KX2-391 | Angiogenesis | Src inhibitor |
| Mitoxantrone | DNA Damage/DNA Repair | Topoisomerase inhibitor |
| Pacritinib (SB1518) | Angiogenesis; Proteases/Proteasome; Chromatin/Epigenetic; Tyrosine Kinase/Adaptors; JAK/STAT signaling; Stem Cells | FLT inhibitor; JAK inhibitor; Tyrosine Kinases inhibitor |
| SB1317(TG-02) hydrochloride | Angiogenesis; Cell Cycle/Checkpoint; Chromatin/Epigenetic; Tyrosine Kinase/Adaptors; JAK/STAT signaling; Stem Cells | CDK inhibitor; FLT inhibitor; JAK inhibitor |


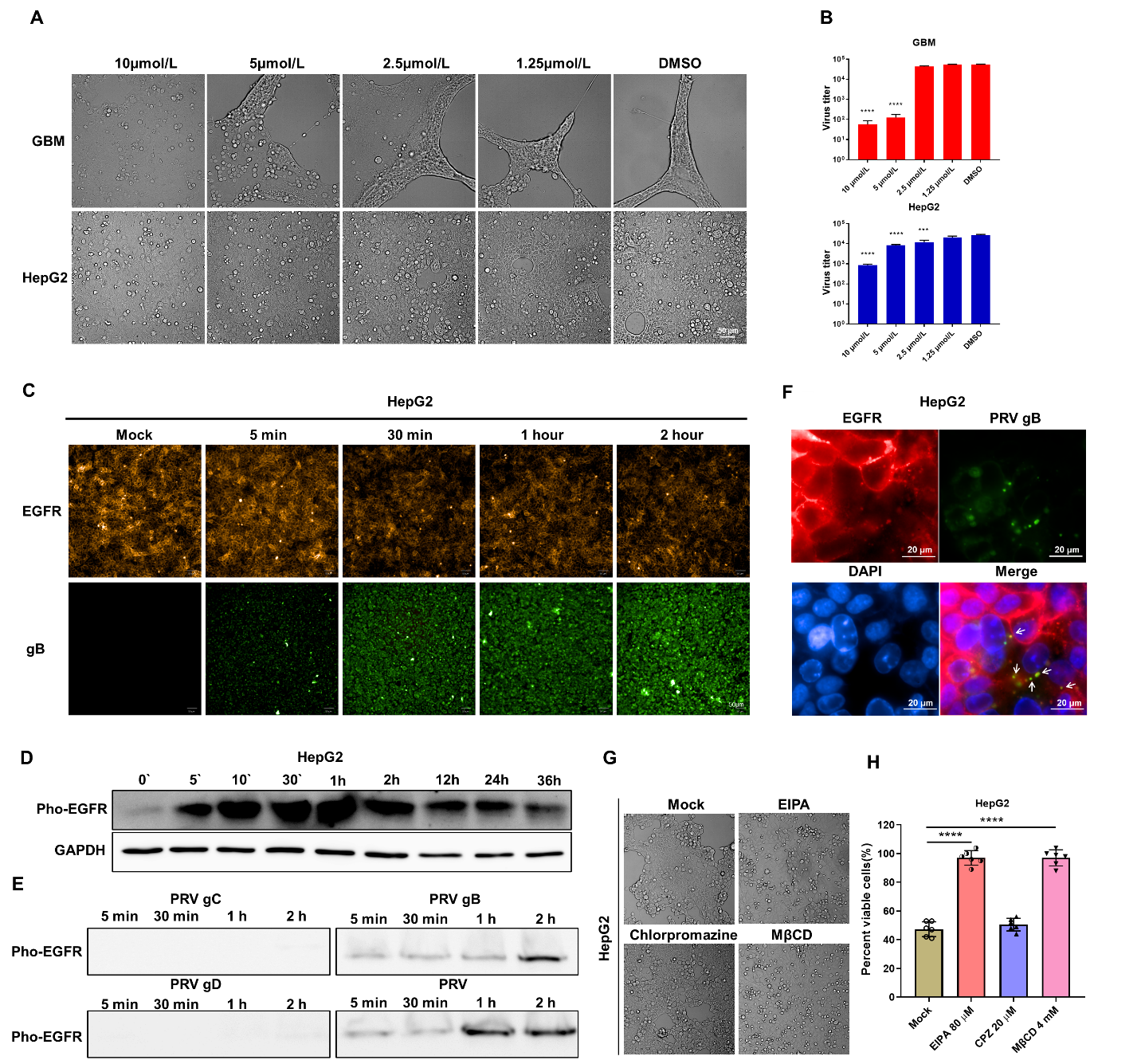
Fig. S1. PRV proliferation is associated with EGFR signaling. (A, B) GBM, HepG2 and Panc-1 cells were pretreated with different concentrations of afatinib or vehicle (DMSO) and were then infected with PRV-LAV. (A) Phase-contrast micrographs. (B) Viral titers in the cell culture supernatant. Scale bar, 50 μm. (C) Immunofluorescence staining of EGFR and PRV gB in HepG2 cells at 0 min, 5 min, 30 min, 1 h and 2 h post-PRV-LAV HB2000 infection. Phase-contrast and fluorescence micrographs were acquired. Scale bar, 50 μm. (D) Western blot analysis of phosphorylated EGFR in serum-starved HepG2 cells at 0 min, 5 min, 10 min, 30 min, 1 h, 2 h, 12 h, 24 h, and 36 h post-PRV-LAV HB2000 infection. GAPDH was used as the loading control. (E) Various PRV glycoproteins expressed in eukaryotic expression system were used to treat serum-starved HepG2 cells for 5 min, 30min, 1h and 2h, and then the expression of phosphorylated EGFR was detected by western blot. (F) The colocalization of EGFR with PRV gB protein at 24 h post-PRV-LAV infection. The white arrows indicate EGFR colocalization with PRV gB. Scale bar, 20 μm. (G-H) PRV-LAV proliferation was suppressed by EIPA and MβCD, but not CPZ. HepG2 cells were pre-incubated with the indicated doses of EIPA, MβCD and CPZ for 30 min, followed by PRV-LAV (MOI = 0.01) infection for 2 h. The cells were then washed with Hanks Balanced Salt Solution twice and cultured for 72 h. Phase-contrast micrographs were acquired (G). The percentage of viable cells was determined (H). Data are presented as the mean ± s.d. values (n = 6). One-way ANOVA was used to determine the significance of differences. *P<0.05; **P<0.01; ***P<0.001; ****P<0.0001.

**
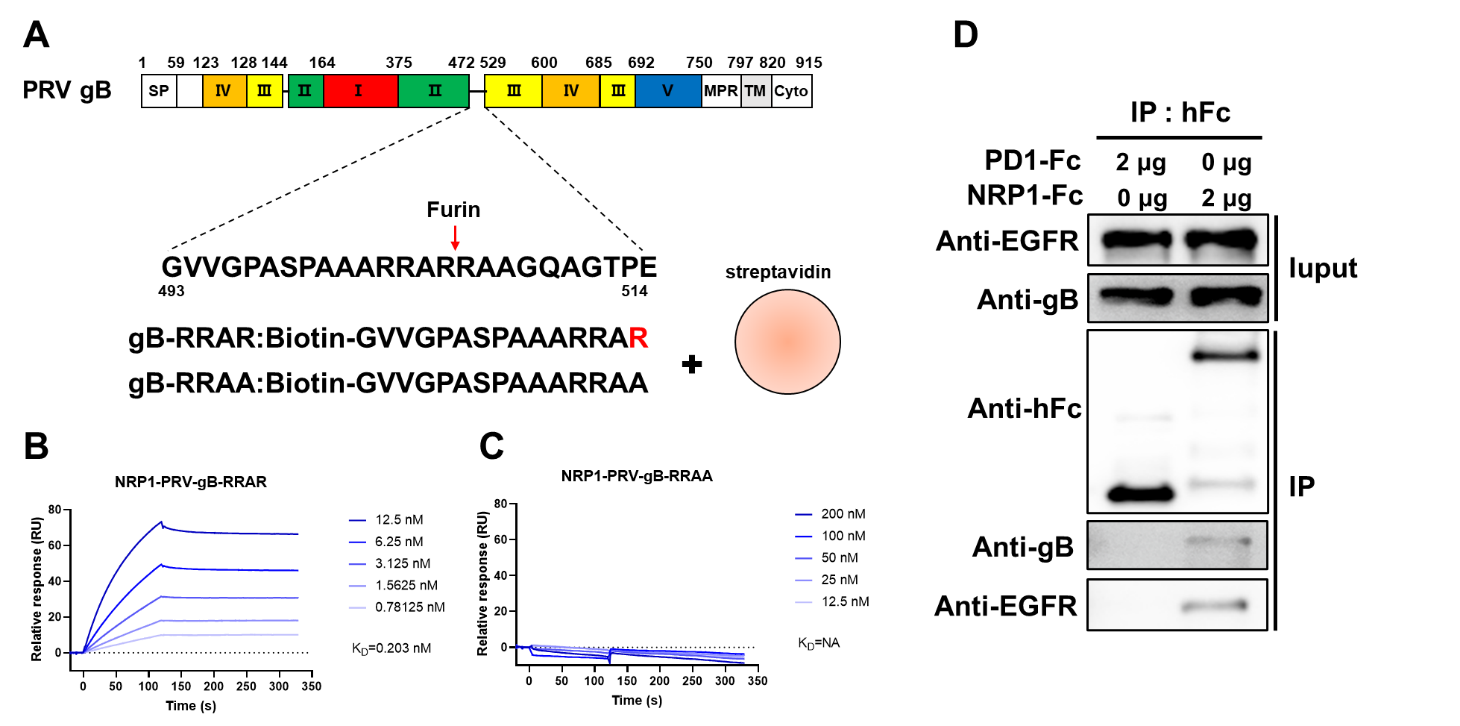
**

**Fig. S2. PRV-LAV gB interacts with EGFR via NRP1.** (**A**) Schematic representation of PRV gB architecture and the peptides containing the PRV gB furin cleavage site. (**B-C**) Affinity between NRP1 and the streptavidin-conjugated PRV peptides, Biotin-gB RRAR (B) and Biotin-gB RRAA (C). Affinity binding was determined using SPR. (**D**) Pull-down assay. Lysates from HepG2 cells infected with PRV-LAV for 48 h were subjected to pull down assay using NRP1. This experiment was repeated three times with similar results; one replicate is represented.


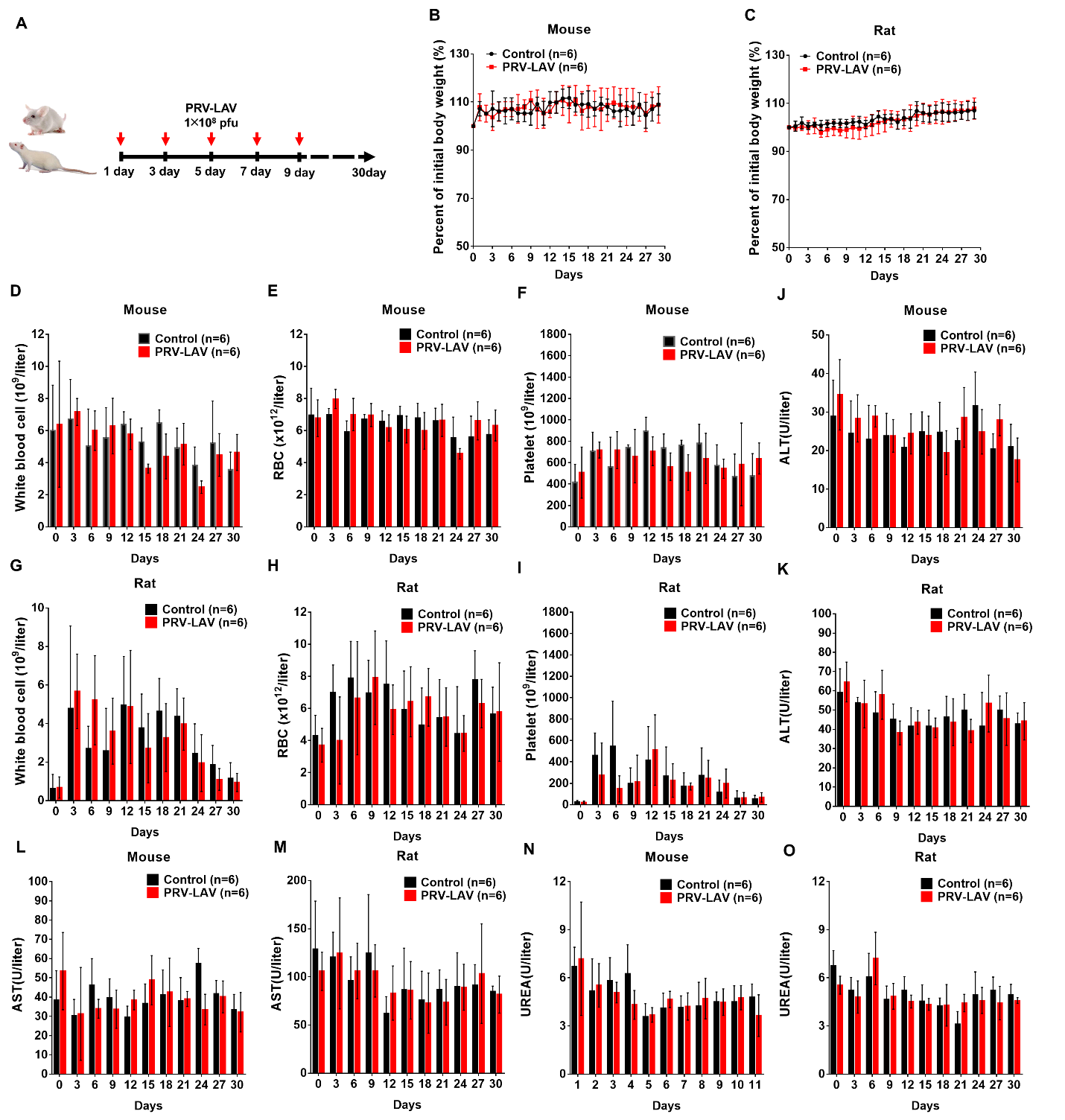


**Fig. S3.** **PRV-LAV treatment** **was well tolerated in mice and rats.** (**A**) Timeline of the experimental setup. (**B**, **C**) Body weight curves for mice (B) and rats (C) treated with vehicle and PRV-LAV HB2000. (**D**-**I**) Hematological parameters of mice (D-F) and rats (G-I) injected with vehicle or PRV-LAV HB2000. RBC, red blood cell. (**J-O**) Serum chemistry parameters for mice (J, L, N) and rats (K, M, O) injected with vehicle or PRV-LAV HB2000. ALT, alanine transaminase; AST, aspartate aminotransferase; UREA, blood urea nitrogen. Data are presented as the mean ± s.d. values (n = 6).

**Table S4. The phenotypes of 19 kinds of clusters.**

| Cluster | Marker | | | Phenotype |  |
| --- | --- | --- | --- | --- | --- |
|  |  |  |  |  |  |
| 1 | | CD45^+^ CD3E^-^ CD19^+^ | B cells | |  |
| 2 | | CD45^+^ CD11b^+^ MHC2^-^ LY6C^+^ LY6G^-^ | Monocytes | |  |
| 3 | | CD45^+^ CD3E^+^ CD8^+^ Tbet^hi^ | activated CD8^+^ T cells | |  |
| 4 | | CD45^+^ CD3E^+^ CD8^+^ PD1^hi^ LAG3^hi^ Tim3^hi^ | exhausted CD8^+^ T cells | |  |
| 5 | | CD11b^+^ LY6C^-^ LY6G^-^ | Macrophages | |  |
| 6 | | CD11b^+^ CD11C^+^ MHC2^+^ | Dendritic cells | |  |
| 7 | | CD45^+^ CD3^-^ NK1.1^+^ | NK cells | |  |
| 8 | | CD11b^+^ LY6C^+^ LY6G^+^ | Neutrophil | |  |
| 9 | | CD45^+^ CD3E^+^ CD4^+^ | CD4^+^ T cells | |  |
| 10 | | CD45^+^ CD11b^+^ MHC2^-^ LY6C^+^ LY6G^-^ | Monocytes | |  |
| 11 | | CD11b^+^ LY6C^+^ LY6G^+^ CD86^+^ | Neutrophil | |  |
| 12 | | CD11b+ MHC2^-^ LY6C^+^ LY6G^-^ | Monocytes | |  |
| 13 | | CD45^+^ CD3E^+^ CD4^-^ CD8^-^ | DN T cells | |  |
| 14 | | CD44^+^ CD45^-^ | Non-lymphocytes | |  |
| 15 | | CD11b^+^ LY6C^+^ LY6G^+^ CD86^-^ | naïve Neutrophil | |  |
| 16 | | CD11b^+^ CD11C^+^ MHC2^+^ | Dendritic cells | |  |
| 17 | | CD11b^+^ LY6C^+^ LY6G^+^ CD86^+^ | activated Neutrophil | |  |
| 18 | | CD45^+^ CD3E^hi^ CD8^+^ PD1^low^ LAG3^low^ Tim3^low^ | effector CD8^+^ T cells | |  |
| 19 | | CD11b^+^ LY6C^+^ LY6G^+^ | Neutrophil | |  |

+, positive; -, negative; hi, high expression; low, low expression.

**Fig. S4.** **Mean** **fluorescence intensity of markers in cluster 3 (activated CD8^+^ T cell).** The mean fluorescence intensity of markers derived from CyTOF analysis of tumor immune infiltrates in Hepa1-6 tissues following vehicle or PRV-LAV treatment. Data are presented as the mean ± s.d. values (n = 3). A t test was used to determine the significance of differences. Red arrowheads indicate those markers with statistically significant differences. *P<0.05; **P<0.01; ***P<0.001; ****P<0.0001.

**Fig. S5. Mean fluorescence intensity of markers in cluster 4 (exhausted CD8^+^ T cell).** The mean fluorescence intensity of markers derived from CyTOF analysis of tumor immune infiltrates in Hepa1-6 tissues following vehicle or PRV-LAV treatment. Data are presented as the mean ± s.d. values (n = 3). A t test was used to determine the significance of differences. Red arrowheads indicate those markers with statistically significant differences. *P<0.05; **P<0.01; ***P<0.001; ****P<0.0001.


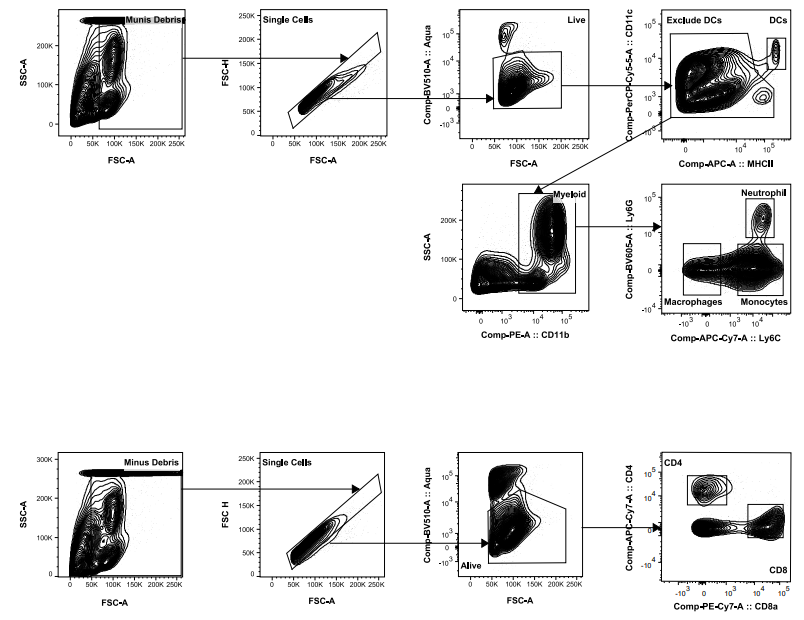


**Fig. S6. The gating strategy of immunological mechanism.**


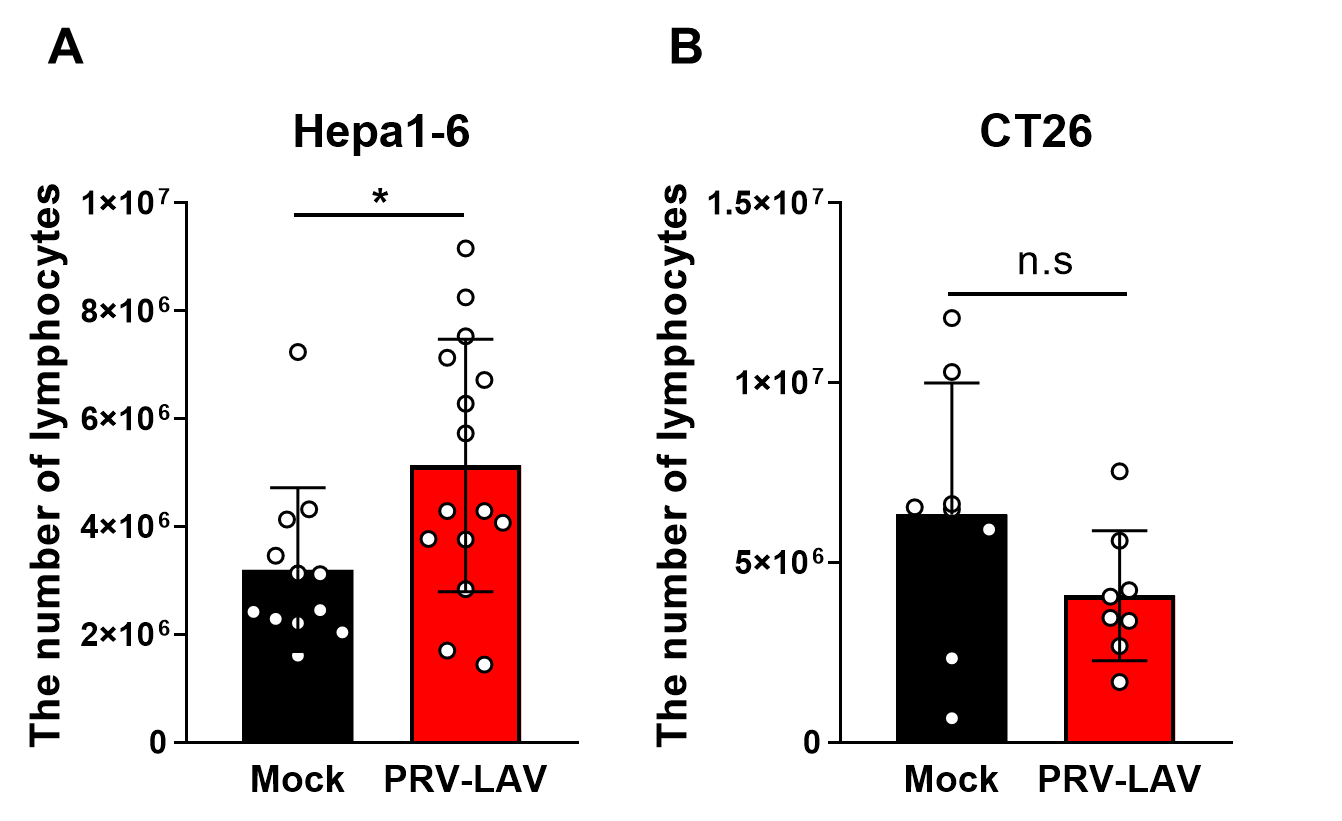
Fig. S7. The total number of lymphocytes in Hepa1-6 and CT26 tumor post mock or PRV-LAV treatment. (A, B) The lymphocytes in Hepa1-6 (A) and CT26 (B) tumor were separated and counted at the day after mice received the final dose of 4 doses of mock (n=12) or PRV-LAV (n=15) treatment. Data are presented as the mean ± s.d. values. The black bars indicate the mean values. A *t* test was used to determine the significance of differences. *P<0.05; **P<0.01; ***P<0.001; ****P<0.0001.
